# Population Genomics Reveals Elevated Inbreeding and Accumulation of Deleterious Mutations in White Raccoon Dogs

**DOI:** 10.3390/biology14010030

**Published:** 2025-01-02

**Authors:** Yinping Tian, Yu Lin, Yue Ma, Jiayi Li, Sunil Kumar Sahu, Jiale Fan, Chen Lin, Zhiang Li, Minhui Shi, Fengping He, Lianduo Bai, Yuan Fu, Zhangwen Deng, Huabing Guo, Haimeng Li, Qiye Li, Yanchun Xu, Tianming Lan, Zhijun Hou, Yanling Xia, Shuhui Yang

**Affiliations:** 1 College of Wildlife and Protected Area, Northeast Forestry University, Harbin 150040, China; yinpingtian@126.com (Y.T.); linyubgi@163.com (Y.L.); mayue@nefu.edu.cn (Y.M.); lijiayi@nefu.edu.cn (J.L.); jialefan@nefu.edu.cn (J.F.); linc110792@nefu.edu.cn (C.L.); b39miku@163.com (L.B.); yuan.fu.99@foxmail.com (Y.F.); lihaimeng66@163.com (H.L.); xu_daniel@163.com (Y.X.); lantianming1314@126.com (T.L.); houzhijundz@163.com (Z.H.); 2 BGI Research, Wuhan 430074, China; sunilkumarsahu@genomics.cn; 3 State Key Laboratory of Agricultural Genomics, BGI-Shenzhen, Shenzhen 518083, China; shiminhui@genomics.cn; 4 College of Life Science, Northeast Forestry University, Harbin 150040, China; 18081077461@163.com (Z.L.); liqiye@genomics.cn (Q.L.); 5 College of Veterinary Medicine, Yunnan Agricultural University, Kunming 650201, China; hefengping@outlook.com; 6 Guangxi Zhuang Autonomous Region Forest Inventory and Planning Institute, Nanning 530011, China; yamasun.dzw817@163.com; 7 Forest Inventory and Planning Institute of Jilin Province, Changchun 130022, China; huabingguo9513@sina.com; 8 Heilongjiang Key Laboratory of Complex Traits and Protein Machines in Organisms, Harbin 150040, China; 9 BGI Life Science Joint Research Center, Northeast Forestry University, Harbin 150040, China

**Keywords:** inbreeding, mutational load, genetic purging, deleterious mutation, ROH, population genomics

## Abstract

The formation of animal breeds usually begins with a small subsample from their ancestral population. Deleterious mutations accumulate in the population under genetic drift, inbreeding, and artificial selection during the development and maintenance of traits desired by humans. White raccoon dogs are among the most popular breeds of farmed raccoon dogs, but white raccoon dogs are more susceptible to disease and have a lower reproductive ability. However, the accumulation of deleterious mutations in this white breed is largely unknown. By analyzing and comparing whole-genome sequencing data from 20 white raccoon dogs and 38 normal raccoon dogs, we detected an increased occurrence of loss-of-function (LoF) mutations in white raccoon dogs compared with normal raccoon dogs. With the finding of a significantly higher dosage of homozygous missense mutations in the white raccoon dog genome, we detected a greater fitness cost in white raccoon dogs. Although a much higher FROH level for ROH fragments longer than 1 Mb has been reported in white raccoon dogs, we did not detect a genetic signal of genetic purging in white raccoon dogs. This study provides valuable genomic resources and new insights into the accumulation of mutation loads in farmed raccoon dogs.

## Simple Summary

Raccoon dogs have been farmed for economic purposes for ~100 years, and white raccoon dogs are among the most famous breeds of farmed raccoon dogs. However, white raccoon dogs are weaker in fitness than normal raccoon dogs. Purging potentially deleterious mutations while maintaining a white coat is the goal for breeders. For the first time, we performed whole-genome sequencing of a white raccoon dog population and performed a comparative analysis with normal raccoon dogs. We found a smaller effective population size for the white raccoon dogs within the last 100 years. The overall inbreeding in the two types of raccoon dogs was found to be very similar, but recently occurring inbreeding was found to be more extensive in white raccoon dogs. Interestingly, the total number of deleterious mutations in the white raccoon dogs is comparable to that in the normal raccoon dogs. However, the proportion of homozygous missense mutations in white raccoon dogs is greater than that in normal raccoon dogs, indicating that the accumulation of small-effect deleterious mutations may be facilitated during the development of white breeds.

## 1. Introduction

Deleterious variants in a population are constantly created with the process of mutation occurring across the genome. These deleterious mutations usually change the function of proteins and further reduce the fitness of species [1]. The burden of deleterious mutations in a population has attracted increasing interest since the middle of the last century and is referred to as the mutational load [2,3]. Many of these deleterious mutations are weakly deleterious but may persist in a population and reduce fitness [4,5]. In contrast, some of these deleterious variants can be quickly eliminated under purifying selection if exposed by homozygosity, particularly mutations with severely damaging effects on fitness in small wild populations [6,7,8,9].

For domesticated animals, the initial founder population usually consists of a small subsample of wild progenitor populations [10]. This domestication bottleneck therefore resulted in a drastically reduced effective population size (*Ne*), and the *Ne* further decreased under subsequent artificial selection for the desired traits [11,12]. The reduced *Ne* could result in a rapid increase in the frequency of deleterious mutations in a population due to strong genetic drift [13]. The artificial selection of favorable human characteristics in domestic animals could also increase the mutational load via the hitchhiking effect [14,15,16]. In addition, relaxed purifying selection under human management could also contribute to the excess accumulation of deleterious mutations, even for large-effect damaging alleles [17,18]. This phenomenon is more significant in some breeds and is usually facilitated by inbreeding. The high frequency of heritable disease in dogs could reflect the increased mutational load due to domestication and the formation of new breeds [19,20]. An excess mutational load in domesticated animals has also been reported in horses, rabbits and cultivated crops [12,21,22]. Purging these deleterious mutations while accelerating breeding is an important goal for breeders [23]. Understanding the mutational load across the genome of a population is vital for the breeding and management of farmed animals.

The raccoon dog (*Nyctereutes procyonoides*) is a typical canid species belonging to the genus *Nyctereutes*, with a number of species-specific biological characteristics, including a diverse diet, high reproductive capacity, winter sleep, and a potentially diverse immune system [24]. The raccoon dog is an omnivore with a very diverse diet, ranging from plants to animals (insects, birds and mammals) [25,26]. This species usually has a large average litter size, with up to 8–10 individuals, and its age at sexual maturity is relatively young [26,27,28,29]. The winter sleep of raccoon dogs can protect the them from cold weather and food deprivation [30]. Unlike hibernation, the body temperature of a raccoon dog is close to normal during winter sleep [30]. Although the racoon dog is distributed across most of Eurasia and is considered a prominent invasive species in Europe, its native distribution is in East Asia [31,32]. Additionally, the raccoon dog is a typical farmed animal and is a famous fur-bearing animal that has been farmed worldwide for ~100 years [26,33]. The winter fur of a raccoon dog is long and thick, and the coat color is usually brownish-gray with black guard hairs, forming a dark stripe on the back (wild-type). Coat color is one of the most important factors contributing to the economic value of the fur coat.

**Figure 1:**
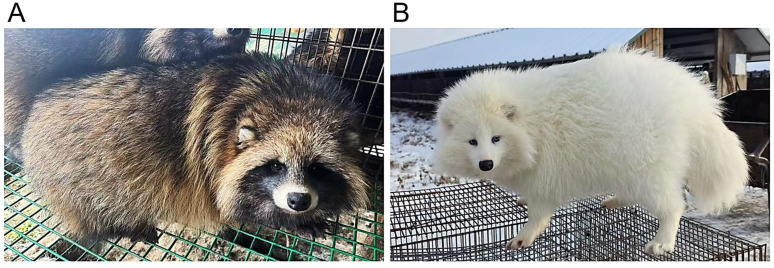
The images of wild-type (**A**) and white-type (**B**) raccoon dogs.

During the long-term breeding of raccoon dogs, several color types other than the wild type have emerged and have been bred on farms. The white coat mutant (white-type) raccoon dog was first identified in the 1970s and was favored the most by people among the different derived color types [34] (Figure 1). Moreover, the quality of the white coat is comparable to that of the coat from wild-type raccoon dogs, making it stand out even more [35]. Therefore, it has a much larger population size and is more common than black-type and yellow-type raccoon dogs. White racoon dogs occur in China and Japan, and are rarely found in the wild. Notably, mating between white-type raccoon dogs is likely to result in stillbirth, and almost all white-type raccoon dogs are hybrids of wild-type raccoon dogs and white-type raccoon dogs, which supports a dominant mode of inheritance for the formation of the white coat in raccoon dogs [36]. In addition, white-type raccoon dogs still have relatively lower reproductive ability [37] and are more susceptible to disease. We hypothesized that the poor fitness of white-type raccoon dogs may stem from the accumulation of deleterious mutations across the genome during the development of this breed, but this phenomenon has never been investigated. Whether inbreeding contributes to the accumulation of mutational load is also unknown. In this study, we performed a population genomic analysis to comprehensively screen the mutational load in white-type raccoon dogs and compared it with that in wild-type individuals, which aimed to improve the breeding of white-type racoon dogs.

## 2. Materials and Methods

### 2.1. Samples and Sequencing Data

Blood samples from a total of 20 white-type raccoon dogs were collected from Harbin Hualong Farm, Harbin, Heilongjiang, China, between 2019 and 2023. These blood samples were collected during routine physical examination and promptly placed into BD Vacutainer Blood Collection Tubes (BD, Franklin Lakes, NJ, USA) for transfer to the laboratory on dry ice. In addition, we downloaded whole-genome resequencing data for 38 wild-type raccoon dogs (collected between 2018 and 2022) from the CNGB Sequence Archive (CNSA) under accession number CNP0002053.

### 2.2. DNA Isolation, Library Preparation, and Sequencing

DNA was extracted from blood samples by using the HiPure Blood DNA Mini Kit (catalog no. D3111) (Magen, Guangzhou, China), according to the manufacturer’s procedures. Qubit 4.0 (Invitrogen, Carlsbad, CA, USA) and agarose gel electrophoresis were used to measure DNA quantity and quality. Qualified DNA (amounts > 300 ng) was then used for DNA library preparation using the MGIEasy Universal DNA Library Preparation Kit (MGI, Shenzhen, China). The libraries with an insert size of ~300 bp to ~500 bp were then subjected to the DNBSEQ T7 sequencer (MGI, Shenzhen, China) for paired-end (100 bp) whole-genome sequencing.

### 2.3. Genome-Wide Variant Calling

We first removed the adapter sequences and low-quality bases from the raw sequencing reads via Trimmomatic [38] (v0.33.0) (Table S1). The filtered clean reads were subsequently aligned to the raccoon dog reference genome [24] via the Burrows–Wheeler Aligner [39] (BWA, version 0.7.10-r789) *mem* algorithm with default parameters. Next, the BAM format alignment files were sorted and deduplicated via Picard tools (http://picard.sourceforge.net) (accessed on 4 March 2016) (version: 2.1.1). A raw variant set was called for each individual by HaplotypeCaller implemented in the Genome Analysis Toolkit [40] (GATK, version: 4.0.3.0), and a Genome Variant Call Format (gVCF) file for each individual was generated. Then, variant joint calling was carried out by combining all gVCF files into a population-based variant call format (VCF) file, which contained both SNPs and InDels. SNPs were then selected for subsequent analysis via GATK with the parameter “SelectVariants–select-type-to-include SNP”.

The SNP set was filtered in a series of steps: (1) Hard filtering was first performed with the parameters “QUAL < 30.0 || QD < 2.0 || FS > 60.0 || MQ < 40.0 || MQRankSum < −12.5 || ReadPosRankSum < −8.0”; (2) SNPs with a sequencing depth in the top and bottom 0.25% were filtered; and 3) those with a missing rate greater than 10% were filtered using VCFtools [41] (version: 0.1.13). This filtered high-quality SNP set was used for population genomic analysis [9].

Before the downstream analysis, we inferred the family relationships among all 58 raccoon dog individuals with KING [42] (version: 2.2.7) to remove potentially closely related individuals (parent–offspring, monozygotic twins, and full siblings). We ultimately retained 34 unrelated individuals, including 21 wild-type individuals and 13 white-type individuals (Table S2). The Perl script “annotate_variation.pl” in ANNOVAR [43] (version: 2015-12-14) software was used for annotating SNPs to identify exotic, nonsynonymous, synonymous, UTR, intronic, intergenic, splicing, and noncoding RNA (ncRNA).

### 2.4. Genetic Structure, Linkage Disequilibrium (LD) Decay, and Genetic Diversity

Here, we performed principal component analysis (PCA), phylogenetic tree construction, and admixture analysis to explore the potential genetic structure of the wild-type and white-type raccoon dog populations. Before this analysis, the SNP set was pruned with VCFtools with the parameter “–thin 1000”. PCA was subsequently carried out by the Genome-wide Complex Trait Analysis [44] (GCTA, version: 1.91.4beta3) software. For the phylogenetic tree, we first converted the VCF file into PHYLIP format by using vcf2phylip [45] (v2.7). We subsequently constructed a maximum-likelihood (ML) phylogenetic tree via IQ-TREE [46] (v 2.3.6) with default parameters. ModelFinder Plus (MFP) was used to identify the best substitution model automatically. Admixture analysis was performed with K values ranging from 2 to 5 via ADMIXTURE [47] (version: 1.3.0). LD decay was inferred for both the white-type and wild-type raccoon dog populations via PopLDdecay (v3.43) software with default parameters. Genome-wide genetic diversity (π) was computed via VCFtools (v 0.1.16) in a nonoverlap 500 kb window with the parameters “vcftools –gzvcf vcf.gz –window-pi 500000 -out result”.

### 2.5. Estimation of Inbreeding by Screening ROHs

ROH fragments in both white-type and wild-type raccoon dogs were detected via PLINK [48] (v1.90b6.10) with the following parameters: “–homozyg –homozyg-window-snp 20 –homozyg-kb 10 –homozyg-density 50” [49,50]. We did not consider ROH fragments shorter than 10 kb in this study. The F_ROH_ was calculated by dividing the total length of ROHs across the autosomes by the total length of the autosomes.

### 2.6. Screening of Genome-Wide Mutational Load

SnpEff [51] (v.5.0e) software was used to identify the mutational load in protein-coding genes. Missense variants annotated by SnpEff [51] were identified as missense variants. Splice acceptors, stops gained, and splice donors annotated by SnpEff [51] were predicted as loss-of-function (LoF) mutations. Deleterious nonsynonymous SNPs (dnsSNPs) with Grantham scores ≥150 were identified [52]. To determine the derived alleles, we downloaded reference genomes for four species from the NCBI: *Vulpes vulpes* (GCF_003160815.1), *Vulpes lagopus* (GCF_018345385.1), *Canis lupus familiaris* (GCF_011100685.1), and *Canis lupus dingo* (GCF_003254725.2). The ancestral allele was determined as the majority allele (occurring at least three times) in the five-species (*V. vulpes*–*V. lagopus*–*C. lupus familiaris–N. procyonoides*) alignment file. The occurrence of mutational load inside ROH and outside ROH regions was estimated by dividing the total number of deleterious mutations in ROH or non-ROH regions by the total number of synonymous mutations in the same genomic regions.

### 2.7. Historical Population Dynamics

We first infer the population history by using MSMC2 [53] with the parameters “-R -i 20 -t 6 -p ‘10*1  +  15*2’”. We randomly selected four individuals from each population for this analysis. Before MSMC computation, we phased the VCF files using BEAGLE (version 5.0) [54] with default parameters, and bamCaller.py was subsequently used to mask the uncovered regions. The final result was visualized with a mutation rate of 1.0 × 10^−8^ substitutions per site per generation and a generation time of 3 years [24]. The change in *Ne* within the most recent 100 generations was analyzed using GONE [55] software (available at https://github.com/esrud/GONE) (accessed on 8 July 2020) with the following parameters: hc = 0.01, NBIN = 667, and cMMb = 1.554.

## 3. Results

### 3.1. Genome-Wide Variant Calling and Identification of Deleterious SNPs

The average sequencing depth of the whole-genome sequencing (WGS) data generated for 20 white raccoon dogs in this study reached 13.9 ± 1.52-fold, and the coverage of the reference genome (Raccoon_dog.p1, CNSA: CNP0002053) reached 97.8% ± 0.23%, which is similar to that of the WGS data for the 38 wild-type raccoon dogs in our previous study [24], with a 13.5 ± 1.18-fold and 98.1 ± 0.41% sequencing depth and coverage, respectively (Table S3). The high-coverage sequencing data [56], associated with the same sequencing platform we used for both the white-type and wild-type samples, could support the subsequent population genomics analysis. By combining sequencing data from all white-type and wild-type raccoon dogs, we detected a total of 8.14 million high-quality SNPs for subsequent analysis after a series of filtering steps. Among these SNPs, 99.3% were distributed in noncoding regions; thus, we focused on the remaining 56,923 SNPs in the coding region to identify deleterious mutations across the genome (Table S4).

**Figure 2:**
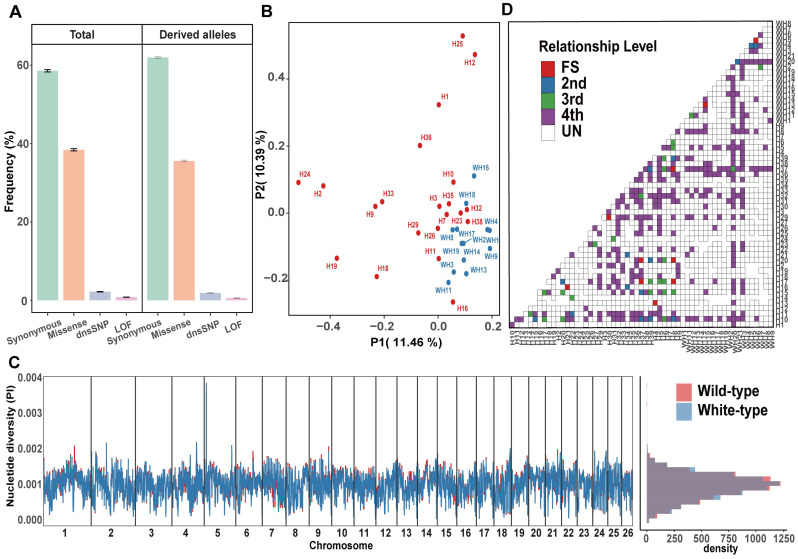
Genetic structure and kinship analysis. (**A**) Comparisons of the frequency of mutational loads for missense, LoF, and dnsSNP in wild-type and white-type raccoon dogs. (**B**) PCA clustering of wild-type and white-type raccoon dogs. (**C**) Genome-wide genetic diversity (π) in nonoverlapping 500 kb windows across the genomes of wild-type and white-type raccoon dogs. (**D**) Relationships among the raccoon dog samples in this study (the inferred relationship types include FS (full siblings), 2nd (2nd-degree relatives), 3rd (3rd-degree relatives), 4th (4th-degree relatives), and UN (unrelated individuals)).

We focused on missense, dnsSNP, and LoF mutations in this study to present the small-effect, moderate-effect, and large-effect mutational loads, respectively. Finally, we identified 23,303 missense mutations, 1379 dnsSNPs, and 561 LoF mutations in the whole population we studied (Table 1). In general, the deleterious mutations in a population usually persist at a relatively low frequency, either for the ancestral or for the derived alleles. In this study, we found that all three types of potentially deleterious mutations maintained a much lower frequency than did the synonymous mutations, with the lowest allele frequency found for LoF mutations followed by dnsSNP and missense mutations (Figure 2A). Compared with mutations in noncoding regions, these deleterious mutations are rarely fixed in the population, and a large proportion of these mutations have a minor allele frequency of less than 0.05 (Figure S1). These characteristics indicate that the three types of mutations identified in this study are truly deleterious [57], which could support the downstream analysis.

### 3.2. Genetic Structure and Kinship Analysis

Although these 58 raccoon dog individuals were collected from a single farm, the origins of these individuals may be very different because the raccoon dogs on a farm are frequently exchanged between different farms to avoid inbreeding depression or to introduce high-quality individuals. Therefore, we first investigated the genetic structure of these individuals to facilitate the subsequent population genomic analysis. The PCA results suggested that the white raccoon dogs did not distinctly separate from the wild-type raccoon dogs, with some individuals overlapping together in the PCA plot (Figure 2B). However, we could still find a blurred border between the two types of raccoon dog populations. The subsequent phylogenetic tree and admixture analysis also revealed similar results, with a blurred border between these two types of raccoon dogs (Figure S2). Although the wild-type raccoon dog population appeared more diverse with a more scattered distribution in the PCA plot, the genetic diversity across the genome was comparable between the two types of raccoon dogs (Figure 2C), largely due to the breeding management at the farm involving importing individuals from other farms worldwide for mating to maintain the health and diversity of the white-type raccoon dog population. Furthermore, we found that 48 pairs of individuals were closely related (Figure 2D), and we removed one individual from each pair to keep unrelated individuals for downstream analysis.

### 3.3. The Accumulation of Deleterious Mutations in White Raccoon Dogs

**Figure 3:**
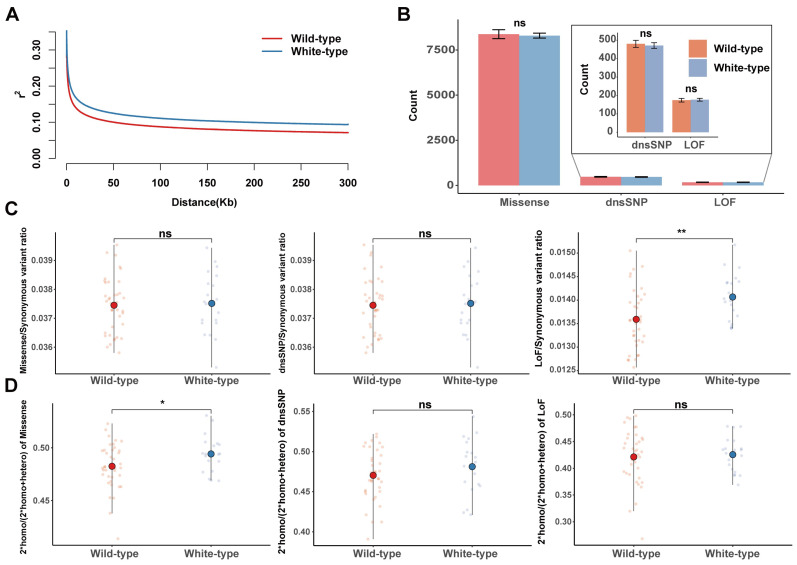
The accumulation of deleterious mutations in raccoon dogs. (**A**) LD decay for the two types of raccoon dog populations. (**B**) Comparisons of the number of mutational loads for missense, LoF, and dnsSNP in wild-type and white-type raccoon dogs. (**C**) Comparisons of the number of mutational loads for missense, LoF, and dnsSNP in wild-type and white-type raccoon dogs, normalized by the number of synonymous mutations in the same region for each individual. (**D**) The ratio of homozygous missense, LoF, and dnsSNP mutations in each individual. “ns” indicates no significant difference, with a *p*-value greater than 0.05. * *p* < 0.05, ** *p* < 0.01.

LD decay was obviously faster in the wild-type raccoon dogs than in the white-type racoon dogs, indicating that stronger hitchhiking may occur in white raccoon dogs (Figure 3A). To explore whether the breeding process of white-type raccoon dogs influences the accumulation of deleterious mutations in the genome, we compared the three types of deleterious mutations across the genome between white-type and wild-type raccoon dogs. Overall, for the total number of deleterious mutations, we did not find a significantly greater mutational load in white-type raccoon dogs than in wild-type raccoon dogs (Figure 3B). We further focused on the occurrence of the mutational load (the number of deleterious mutations divided by the number of synonymous mutations) to reduce the bias introduced from the different genetic backgrounds between these two populations. We found a significantly elevated occurrence of LoF mutations in the white raccoon dog population, although this obvious difference was not found for the missense and dnsSNP mutations (Figure 3C). This finding indicated that the proportion of large-effect deleterious mutations in white raccoon dogs was much greater than that in wild-type raccoon dogs. The homozygous deleterious mutation usually represents the exposed mutational load that has a direct negative impact on fitness. In this study, we did not find a significant difference in the number of homozygous genotypes with different mutational loads between white-type raccoon dogs and wild-type raccoon dogs (Figure S3). However, white raccoon dogs presented a significantly elevated allele dosage of homozygous missense mutations but not dnsSNP or LoF mutations (Figure 3D).

### 3.4. Inbreeding Facilitated the Accumulation of Deleterious Mutations

**Figure 4:**
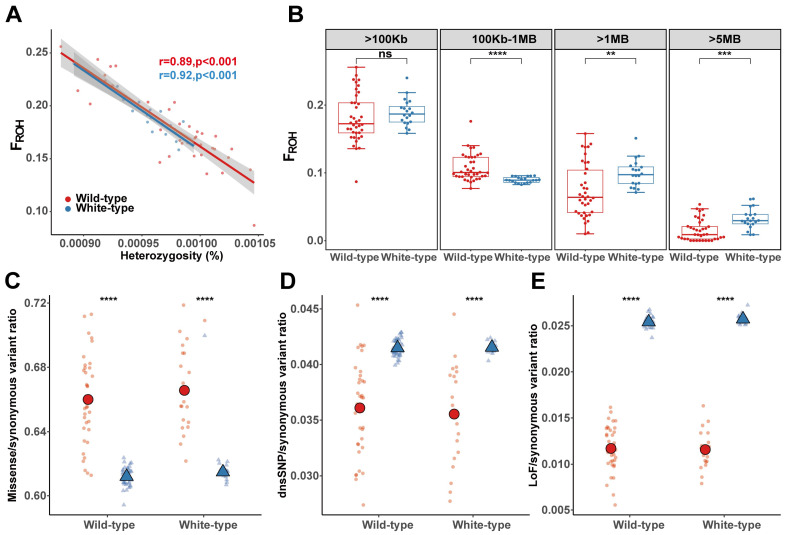
ROH and mutational load in two raccoon dog populations. (**A**) Relationships between the F_ROH_ and heterozygosity in wild-type and white-type raccoon dogs. (**B**) Distribution of F_ROH_ for different ROH lengths in wild-type and white-type raccoon dog populations. (**C**) The distribution of missense mutations in ROH regions and outside ROH regions, normalized by the number of synonymous mutations in the same region for each individual. (**D**) The distribution of dnsSNP mutations in ROH regions and outside ROH regions, normalized by the number of synonymous mutations in the same region for each individual. (**E**) The distribution of LoF mutations in ROH regions and outside ROH regions, normalized by the number of synonymous mutations in the same region for each individual. The blue triangles represent “outside ROH regions” and the red circles represent “inside ROH regions” in (**C**–**E**). “ns” indicates no significant difference, with a *p*-value greater than 0.05. ** *p* < 0.01, *** *p* < 0.001, **** *p* < 0.0001.

Although the genetic diversity of wild-type raccoon dogs was comparable to that of white-type raccoon dogs (Figure S4), we explored whether repeated mating among white raccoon dogs results in an elevated inbreeding level by screening for ROHs across the genome and comparing the results with those of wild-type raccoon dogs. Overall, genome heterozygosity was negatively related to F_ROH_ in both types of raccoon dogs (Figure 4A), indicating the contribution of inbreeding to the decrease in genome-wide genetic diversity. By further examining the ROH distribution, we found that the lengths of most of the ROHs were restricted to less than 1 Mb in the raccoon dog population (Figure S5), which accounted for 92.03% of the total number of ROH fragments. This finding indicated that recent inbreeding events (the most recent 3–5 generations) in farmed raccoon dogs may not have been frequent. Nonetheless, the overall F_ROH_ for ROH greater than 100 kb in the farmed raccoon dog population still reached 18.38%, and no significant differences were found between white-type raccoon dogs (F_ROH_ = 18.91%) and wild-type raccoon dogs (F_ROH_ = 18.11%) (Figure 4B). Interestingly, we detected a significantly lower inbreeding level in white racoon dogs when ROH fragments shorter than 1 Mb were considered. However, a significantly greater level of inbreeding in white-type raccoon dogs was detected when we focused on ROH fragments longer than 1 Mb and 5 Mb (Figure 4B).

We subsequently explored the impact of recent inbreeding on the accumulation of deleterious mutations across the genome in white racoon dogs. We found a very significantly elevated occurrence of missense mutations in the ROH regions in both the wild-type and white-type individuals, but the occurrence was greater in the white racoon dogs (Figure 4C). However, the occurrence of LoFs and dnsSNPs in the ROH region presented a decreasing trend inside the ROH regions compared with outside the ROH regions (Figure 4D,E). This may be explained by the fact that large-effect deleterious mutations are much easier to purge by purifying selection when exposed by inbreeding; therefore, the increasing trend of the accumulation of deleterious mutations could hardly be detected.

### 3.5. Population Dynamics Analysis

**Figure 5:**
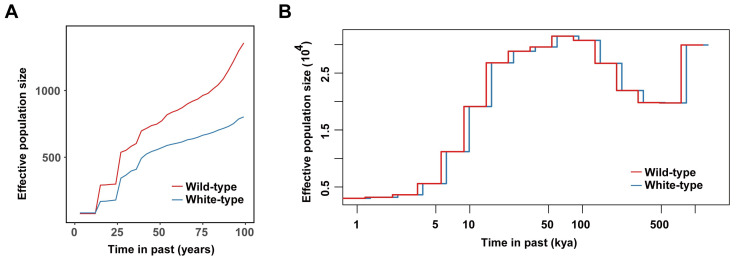
Population history of the two raccoon dog populations. (**A**) Recent effective population size *(Ne*) changes inferred by the GONE. (**B**) The dynamic trajectory of the *Ne* of the two types of raccoon dogs inferred by MSMC2.

Historical population dynamics are also key factors that impact the accumulation of deleterious mutations. Here, we first examined the dynamics of the effective population sizes of these two types of raccoon dogs over their entire evolutionary history. As we expected, we found a very similar population history for white-type raccoon dogs to that of the wild-type population from 100 thousand years ago (kya) to 1 kya (Figure 5A), which was very consistent with our previous findings of two population contraction events and one population expansion event [24]. Considering the short breeding history of raccoon dogs (~100 years and ~50 years for wild-type and white-type raccoon dogs, respectively), we focused on their population history within the most recent 100 years (Figure 5B). Both types of raccoon dogs presented an overall decreasing trend during this period; however, white raccoon dogs had a much smaller effective population than the wild-type raccoon dogs did during this period, which might be explained by the limited number of founders of white-type racoon dogs.

### 3.6. Potentially Deleterious Mutations Associated with the Formation of White Coat Color

Considering that the overall accumulation of deleterious mutations was similar between the two types of raccoon dogs, we focused on private deleterious mutations in white raccoon dogs to explore the functional impact of these mutations on their survival. Unfortunately, we cannot find a genotype that is completely exclusive to white raccoon dogs, indicating that the formation of the white breed has not widely influenced the genetic background of the population or that repeat mating with wild-type raccoon dogs does not allow for the fixation of complete white raccoon dog genotypes.

It was supposed that many deleterious mutations arise with the pursuit of breeding for the white coat phenotype. We screened deleterious mutations that were distributed within and around the genes related to the white coat. Here, we selected 40 genes that were reported to be responsible for the formation of coat color, including the migration of melanocytes, the formation of melanosomes, the synthesis of eumelanin and pheomelanin, and other processes related to the formation of coat color (Table S5). In total, we discovered 25 deleterious mutations in these regions, including 24 missense mutations and one dnsSNP (Table S6). However, none of these mutations had an allele frequency higher than 0.5, indicating that hitchhiking does not significantly influence the accumulation of deleterious mutations during the breeding of white-type breeds, or that the white coat in raccoon dogs may be caused by mutations in more than one gene and that some genes are included in these 40 genes. These 25 deleterious mutations were distributed in four gene regions, including the *AHCY*, *RALY*, *OCA2,* and *TRPM7* genes, and 13 missense mutations were distributed in the *AHCY* gene. All four genes are responsible for the formation of hair or skin color in domesticated animals (Table S7).

## 4. Discussion

In domesticated animals, the initial population usually originates from a small number of individuals from their wild progenitor populations [10]. Different breeds are formed from a further small subsample of the domesticated animals. The white-type raccoon dog is a different breed of farmed raccoon dog [34], which is expected to have a smaller effective population size. In this study, the *Ne* values of the two types of raccoon dogs presented very similar dynamic histories from 100 kya to 1.0 kya, and the difference in the *Ne* values could be detected only between the two types of racoon dogs until the last 100 years. This is consistent with the fact that white raccoon dogs were produced several decades ago and originated from wild-type raccoon dogs. Although the same ancestry was shared by these two types of raccoon dogs, the white-type and wild-type raccoon dogs in this study presented a low level of separation (Figure 2B and Figure S2), which could be explained by the fact that the white-type raccoon dog originated from a subsample of the wild-type raccoon dog population, and this drift resulted in genetic differentiation at the beginning of the formation of this breed; however, repeated mating with the wild-type breed, to some extent, eroded this genetic differentiation. Notably, the limited sample size in this study may have generated a biased structural analysis due to the limited statistical power to detect subtle genetic differences between populations, which could lead to an underestimation of the genetic divergence between white-type and wild-type raccoon dogs.

For domesticated animals, inbreeding seems to be inevitable [14] and would be even more serious for some breeds [58,59]. The inbreeding level (F_ROH_ ≈ 19%) of farmed raccoon dogs is even higher than that of some endangered species [7,60,61] and could be explained by selective mating for the breeding of phenotypes desired by humans. ROH fragments longer than 1 Mb accounted for an extremely small proportion (less than 2%) of the raccoon dog genome (Figure 4A), which was far less than that of other domesticated mammals, such as dog [62], pig [63], and cattle [64] breeds. The total number of breeding generations is no more than 40 generations, considering a generation interval of 3 years for raccoon dogs. The breeding history of raccoon dogs is far shorter than that of other domesticated animals, which could explain the large proportion of ROH fragments shorter than 1 Mb. This is also a genetic signal that 100 years of breeding does not lead to serious inbreeding in farmed raccoon dogs. In contrast, for other long-term domesticated animals, the proportions of ROH longer than 1 Mb are much higher than that in raccoon dogs [62,63,64]. Interestingly, we still detected an obviously elevated inbreeding level for ROHs longer than 1 Mb in white-type racoon dogs (Figure 4A). This is not surprising because (1) the domestication bottleneck for white-type raccoon dogs is much more severe than that for wild-type populations, with only several founders at the beginning of breeding; (2) repeated mating with white-type raccoon dogs is needed to maintain the white coat in offspring; and (3) the breeding history of white-type raccoon dogs is much shorter than that of wild-type raccoon dogs.

The number of de novo deleterious mutations is expected to be almost equal to the number of deleterious mutations purged from the population by purifying selection under population genetics theory [65]. For the breeding of farmed animals, particularly for the maintenance of breeds for desired human phenotypes, however, the frequency of deleterious mutations could be elevated by genetic drift, inbreeding, and artificial selection [22]. Here, we revealed a greater occurrence of LoF across the genome in the white raccoon dogs than that of the wild-type raccoon dogs. This is well in line with many domesticated animals under the theory that the domestication process results in strong genetic drift and the relaxation of purifying selection, generating an elevated accumulation of deleterious mutations in the population, which is also called the “cost of domestication” hypothesis [66]. Whole-genome sequencing analysis revealed a significantly elevated mutation load in domesticated yaks than in wild yaks [57]. An increased proportion of nonsynonymous mutations was also found in dog, rabbit, pig, silkworm, and chicken, as well as in some crops (soybean and rice) [22,57]. However, the occurrence of LoF mutations in the white-type raccoon dogs was lower inside ROH regions than that outside ROH regions, indicating the possible purging of deleterious large-effect LoF mutations. Interestingly, we detected an increased frequency of missense mutations inside ROH regions in white-type raccoon dogs compared with wild-type raccoon dogs. One possibility could explain this phenomenon: purifying selection against deleterious mutations has been relaxed during the formation of white-type raccoon dogs, and the frequency of small-effect deleterious mutations, such as missense mutations, could increase with increasing frequency facilitated by inbreeding under the hitchhiking effect but could not be effectively purged from the population under human care. In wild animals, the accumulation of mutational load is usually negatively related to the inbreeding level (F_ROH_) [6,9], because these homozygous deleterious mutations could be quickly purged in the wild environment. For farmed animals, even these small-effect deleterious mutations could be amplified if these animals are exposed to the wild environment or subjected to poor human care, increasing the potential risk of the survival of raccoon dogs. Intriguingly, we did not find any high-frequency (>50%) deleterious mutations in 40 coat color formation-related genes, which suggests that the deleterious mutations did not increase simultaneously with the artificial selection of the white coat. However, another possibility is that the genetic basis for coat color in raccoon dogs may not stem from currently known genes [67,68], which requires further research for confirmation.

The elevated inbreeding level during the recent breeding history of the farmed raccoon dogs is associated with the increased accumulation of deleterious mutations in the ROH genomic regions, suggesting that breeders should begin to pay attention to balancing inbreeding with the maintenance of excellent fur phenotypes in future breeding programs. Although the large-effect deleterious mutations could be purged from the population, the purging effect has been largely discounted under artificial selection, and the accumulation of small-effect and moderate-effect mutations is also a potential risk for the survival of raccoon dogs. Therefore, purging potentially deleterious mutations while maintaining the human-desired phenotypes in the coat could be a long journey for breeders to pursue.

## 5. Conclusions

A comprehensive investigation of genome-wide inbreeding and accumulation of deleterious effects in domesticated animals is vital for breeding. Balancing the trade-off between the accumulation of deleterious alleles and the pursuit of phenotypes desired by humans is an important task for breeders. In this study, we found similar but different genetic backgrounds between white-type and wild-type raccoon dogs, which could be explained by drift and repeated inbreeding during the formation of the white breed. We further detected more severe inbreeding in white raccoon dogs when ROH fragments longer than 1 Mb were considered, indicating more intensive recent inbreeding in the white breed. We did not find a significant difference in the total number of deleterious mutations between the white-type and wild-type raccoon dogs. However, an increased occurrence of LoF mutations and an increased dosage of homozygous missense mutations were detected in the white racoon dog. In addition, we did not find a favorable accumulation of deleterious mutations in genes that are currently known to be responsible for the formation of coat colors.

The inescapable limitation of this study is the small sampling size, which may influence our results in several aspects, e.g., unbalanced sampling may result in the absence of some representative individuals, which could further lead to biased analysis results; the small sample size could lower the power to detect the fine-scale genetic structure by missing large amounts of low-frequency variants in the population, and then bias the detection of the genome-wide distribution of deleterious mutations. In particular, the small sample size has very limited power in the GWAS analysis, which makes it difficult to detect the genuine causal variants of white coat formation in raccoon dogs. Exploring the genetic basis of coat color beyond the currently known genes in the raccoon dog genome, which would be based on a large sample size, is an interesting direction for future research.

## Figures and Tables

**Table 1:** Distribution of SNPs within the genomic regions of raccoon dog.

SNP Type	SNP Count in Wild-Type Raccoon Dogs	SNP Count in White-Type Raccoon Dogs
Intergenic region	2,248,754 ± 65,291	2,245,684 ± 36,736
Intron variant	888,747 ± 26,938	882,183 ± 14,448
Upstream gene variant	108,076 ± 3393	106,533 ± 1856
Downstream gene variant	105,060 ± 3222	104,215 ± 1583
Synonymous variant	12,825 ± 404	12,560 ± 234
Missense variant	8375 ± 247	8295 ± 137
LoF variant	174 ± 10	177 ± 8
dnsSNP variant	480 ± 19	471 ± 15
Splice acceptor variant	38 ± 3	41 ± 4
Splice donor variant	48 ± 4	49 ± 5
Splice region variant	2007 ± 67	1994 ± 31
Start lost	8 ± 2	8 ± 2
Stop_lost	6.76 ± 1.63	7 ± 1
Stop gained	77 ± 6.41	76 ± 7
Stop retained variant	4.92 ± 1	5 ± 1

## Data Availability

The data that support the findings of this study have been deposited in the China National GenBank at https://www.ncbi.nlm.nih.gov with the accession number CNP0006567.

## Supplementary Materials

The following supporting information can be downloaded at https://www.mdpi.com/article/10.3390/biology14010030/s1: Figure S1. Site-frequency spectrum for deleterious variants (missense, LoF, and dnsSNP mutations combined) and neutral variants (intergenic mutations) for the raccoon dog populations. The number of loci (y axis) is shown for each allele frequency (x axis); Figure S2. (A) Maximum-likelihood phylogenetic tree of raccoon dog populations and (B) admixture analysis results based on K from 2 to 5 for the raccoon dog population; Figure S3. The number of homozygous genotypes of mutational load between the white-type raccoon dogs and wild-type raccoon dogs; Figure S4. The genetic diversity between wild-type and white-type raccoon dogs; Figure S5. The number of homozygous genotypes of ROH length between the white-type raccoon dogs and wild-type raccoon dogs; Table S1. Summary of software used in the study; Table S2. Results of kinship coefficients and propIBD estimation with the KING program; Table S3. Information and statistics of the whole-genome resequencing data of 58 raccoon dogs; Table S4. Summary of the SNP sites; Table S5. Summary of functions of color-related genes; Table S6. A summary of deleterious mutations in color-related gene region; Table S7. Summary of functions of significantly differential color-related genes. Refs. [38,39,40,43,44,45,46,48,51,53,54,69,70,71,72,73,74,75,76,77,78,79,80,81,82,83,84,85,86,87,88,89,90,91,92,93,94,95,96,97,98,99,100,101,102,103,104,105,106,107,108,109,110,111,112,113,114,115,116,117,118,119,120,121,122,123,124,125] are cited in the Supplementary Materials.

## References

[1] Adzhubei I.A., Schmidt S., Peshkin L., Ramensky V.E., Gerasimova A., Bork P., Kondrashov A.S., Sunyaev S.R. (2010). A method and server for predicting damaging missense mutations. Nat. Methods.

[2] Kimura M., Maruyama T., Crow J.F. (1963). The Mutation Load in Small Populations. Genetics.

[3] Ohta T. (1973). Slightly deleterious mutant substitutions in evolution. Nature.

[4] Kono T.J., Fu F., Mohammadi M., Hoffman P.J., Liu C., Stupar R.M., Smith K.P., Tiffin P., Fay J.C., Morrell P.L. (2016). The Role of Deleterious Substitutions in Crop Genomes. Mol. Biol. Evol..

[5] Eyre-Walker A., Woolfit M., Phelps T. (2006). The distribution of fitness effects of new deleterious amino acid mutations in humans. Genetics.

[6] Dussex N., Van Der Valk T., Morales H.E., Wheat C.W., Díez-del-Molino D., Von Seth J., Foster Y., Kutschera V.E., Guschanski K., Rhie A. (2021). Population genomics of the critically endangered kākāpō. Cell Genom..

[7] Khan A., Patel K., Shukla H., Viswanathan A., van der Valk T., Borthakur U., Nigam P., Zachariah A., Jhala Y.V., Kardos M. (2021). Genomic evidence for inbreeding depression and purging of deleterious genetic variation in Indian tigers. Proc. Natl. Acad. Sci. USA.

[8] Kleinman-Ruiz D., Lucena-Perez M., Villanueva B., Fernandez J., Saveljev A.P., Ratkiewicz M., Schmidt K., Galtier N., Garcia-Dorado A., Godoy J.A. (2022). Purging of deleterious burden in the endangered Iberian lynx. Proc. Natl. Acad. Sci. USA.

[9] Lan T., Yang S., Li H., Zhang Y., Li R., Sahu S.K., Deng W., Liu B., Shi M., Wang S. (2024). Large-scale genome sequencing of giant pandas improves the understanding of population structure and future conservation initiatives. Proc. Natl. Acad. Sci. USA.

[10] Lu J., Tang T., Tang H., Huang J., Shi S., Wu C.I. (2006). The accumulation of deleterious mutations in rice genomes: A hypothesis on the cost of domestication. Trends Genet. TIG.

[11] Bosse M., Megens H.J., Derks M.F.L., de Cara A.M.R., Groenen M.A.M. (2019). Deleterious alleles in the context of domestication, inbreeding, and selection. Evol. Appl..

[12] Dwivedi S.L., Heslop-Harrison P., Spillane C., McKeown P.C., Edwards D., Goldman I., Ortiz R. (2023). Evolutionary dynamics and adaptive benefits of deleterious mutations in crop gene pools. Trends Plant Sci..

[13] Charlesworth B. (2009). Fundamental concepts in genetics: Effective population size and patterns of molecular evolution and variation. Nat. Rev. Genet..

[14] Marsden C.D., Ortega-Del Vecchyo D., O’Brien D.P., Taylor J.F., Ramirez O., Vila C., Marques-Bonet T., Schnabel R.D., Wayne R.K., Lohmueller K.E. (2016). Bottlenecks and selective sweeps during domestication have increased deleterious genetic variation in dogs. Proc. Natl. Acad. Sci. USA.

[15] Chun S., Fay J.C. (2011). Evidence for hitchhiking of deleterious mutations within the human genome. PLoS Genet..

[16] Hartfield M., Otto S.P. (2011). Recombination and hitchhiking of deleterious alleles. Evol. Int. J. Org. Evol..

[17] Wiener P., Wilkinson S. (2011). Deciphering the genetic basis of animal domestication. Proc. Biol. Sci..

[18] Wang Z., Yonezawa T., Liu B., Ma T., Shen X., Su J., Guo S., Hasegawa M., Liu J. (2011). Domestication relaxed selective constraints on the yak mitochondrial genome. Mol. Biol. Evol..

[19] Ostrander E.A., Kruglyak L. (2000). Unleashing the canine genome. Genome Res..

[20] Karlsson E.K., Lindblad-Toh K. (2008). Leader of the pack: Gene mapping in dogs and other model organisms. Nat. Rev. Genet..

[21] Schubert M., Jonsson H., Chang D., Der Sarkissian C., Ermini L., Ginolhac A., Albrechtsen A., Dupanloup I., Foucal A., Petersen B. (2014). Prehistoric genomes reveal the genetic foundation and cost of horse domestication. Proc. Natl. Acad. Sci. USA.

[22] Makino T., Rubin C.J., Carneiro M., Axelsson E., Andersson L., Webster M.T. (2018). Elevated Proportions of Deleterious Genetic Variation in Domestic Animals and Plants. Genome Biol. Evol..

[23] Renaut S., Rieseberg L.H. (2015). The Accumulation of Deleterious Mutations as a Consequence of Domestication and Improvement in Sunflowers and Other Compositae Crops. Mol. Biol. Evol..

[24] Lan T., Li H., Yang S., Shi M., Han L., Sahu S.K., Lu Y., Wang J., Zhou M., Liu H. (2022). The chromosome-scale genome of the raccoon dog: Insights into its evolutionary characteristics. iScience.

[25] Drygala F., Werner U., Zoller H. (2013). Diet composition of the invasive raccoon dog (*Nyctereutes procyonoides*) and the native red fox (*Vulpes vulpes*) in north-east Germany. Hystrix.

[26] Kauhala K., Kowalczyk R. (2011). Invasion of the raccoon dog Nyctereutes procyonoides in Europe: History of colonization, features behind its success, and threats to native fauna. Curr. Zool..

[27] Helle E., Kauhala K. (1995). Reproduction in the Raccoon Dog in Finland. J. Mammal..

[28] Kauhala K. (1996). Reproductive strategies of the racoon dog and the red fox in Finland. Acta Theriol..

[29] Kowalczyk R., Zalewski A., Jędrzejewska B., Ansorge H., Bunevich A.N. (2009). Reproduction and Mortality of Invasive Raccoon Dogs (*Nyctereutes procyonoides*) in the Białowieża Primeval Forest (Eastern Poland). Ann. Zool. Fenn..

[30] Asikainen J., Mustonen A.M., Hyvarinen H., Nieminen P. (2004). Seasonal physiology of the wild raccoon dog (*Nyctereutes procyonoides*). Zool. Sci..

[31] Mulder J. (2012). A review of the ecology of the raccoon dog (*Nyctereutes procyonoides*) in Europe. Lutra.

[32] Pitra C., Schwarz S., Fickel J. (2009). Going west—Invasion genetics of the alien raccoon dog Nyctereutes procynoides in Europe. Eur. J. Wildl. Res..

[33] Yan S.Q., Li Y.M., Bai C.Y., Ding X.M., Li W.J., Hou J.N., Zhao Z.H., Sun J.H. (2013). Development and characterization of polymorphic microsatellite markers for Chinese raccoon dog (*Nyctereutes procyonoides procyonoides*). Genet. Mol. Res. GMR.

[34] Du Z., Huang K., Zhao J., Song X., Xing X., Wu Q., Zhang L., Xu C. (2017). Comparative Transcriptome Analysis of Raccoon Dog Skin to Determine Melanin Content in Hair and Melanin Distribution in Skin. Sci. Rep..

[35] Guo Y., Xing X., Wu Q., Xu C., Zhao J. (2019). Production Performance Testing of Wusuli Raccoon Dog with White Mutant Coat. J. Domest. Anim. Ecol..

[36] He R., He Y., Meng Y., Zhao J. (2016). Research Progress on Genetics of Wusuli Racoon Dog of Three Kinds of Color Type (wild type, white type, red brown type). J. Jilin Agric. Sci. Technol. Univ..

[37] Shi G. (2008). How to select the breeding source of white raccoon dog. Spec. Econ. Anim. Plants.

[38] Bolger A.M., Lohse M., Usadel B. (2014). Trimmomatic: A flexible trimmer for Illumina sequence data. Bioinformatics.

[39] Li H. (2013). Aligning sequence reads, clone sequences and assembly contigs with BWA-MEM. arXiv.

[40] McKenna A., Hanna M., Banks E., Sivachenko A., Cibulskis K., Kernytsky A., Garimella K., Altshuler D., Gabriel S., Daly M. (2010). The Genome Analysis Toolkit: A MapReduce framework for analyzing next-generation DNA sequencing data. Genome Res..

[41] Danecek P., Auton A., Abecasis G., Albers C.A., Banks E., DePristo M.A., Handsaker R.E., Lunter G., Marth G.T., Sherry S.T. (2011). The variant call format and VCFtools. Bioinformatics.

[42] Manichaikul A., Mychaleckyj J.C., Rich S.S., Daly K., Sale M., Chen W.M. (2010). Robust relationship inference in genome-wide association studies. Bioinformatics.

[43] Wang K., Li M., Hakonarson H. (2010). ANNOVAR: Functional annotation of genetic variants from high-throughput sequencing data. Nucleic Acids Res..

[44] Yang J., Lee S.H., Goddard M.E., Visscher P.M. (2011). GCTA: A tool for genome-wide complex trait analysis. Am. J. Hum. Genet..

[45] Ortiz E.M. (2019). vcf2phylip v2.0: Convert a VCF Matrix into Several Matrix Formats for Phylogenetic Analysis. Zenodo Geneva. https://zenodo.org/records/2540861.

[46] Nguyen L.-T., Schmidt H.A., Von Haeseler A., Minh B.Q. (2015). IQ-TREE: A fast and effective stochastic algorithm for estimating maximum-likelihood phylogenies. Mol. Biol. Evol..

[47] Alexander D.H., Novembre J., Lange K. (2009). Fast model-based estimation of ancestry in unrelated individuals. Genome Res..

[48] Purcell S., Neale B., Todd-Brown K., Thomas L., Ferreira M.A., Bender D., Maller J., Sklar P., De Bakker P.I., Daly M.J. (2007). PLINK: A tool set for whole-genome association and population-based linkage analyses. Am. J. Hum. Genet..

[49] Dobrynin P., Liu S., Tamazian G., Xiong Z., Yurchenko A.A., Krasheninnikova K., Kliver S., Schmidt-Küntzel A., Koepfli K.-P., Johnson W. (2015). Genomic legacy of the *African cheetah*, *Acinonyx jubatus*. Genome Biol..

[50] Meyermans R., Gorssen W., Buys N., Janssens S. (2020). How to study runs of homozygosity using PLINK? A guide for analyzing medium density SNP data in livestock and pet species. BMC Genom..

[51] Cingolani P., Platts A., Wang L.L., Coon M., Nguyen T., Wang L., Land S.J., Lu X., Ruden D.M. (2012). A program for annotating and predicting the effects of single nucleotide polymorphisms, SnpEff: SNPs in the genome of Drosophila melanogaster strain w1118; iso-2; iso-3. Fly.

[52] Grantham R. (1974). Amino acid difference formula to help explain protein evolution. Science.

[53] Schiffels S., Durbin R. (2014). Inferring human population size and separation history from multiple genome sequences. Nat. Genet..

[54] Browning B.L., Zhou Y., Browning S.R. (2018). A One-Penny Imputed Genome from Next-Generation Reference Panels. Am. J. Hum. Genet..

[55] Santiago E., Novo I., Pardiñas A.F., Saura M., Wang J., Caballero A. (2020). Recent demographic history inferred by high-resolution analysis of linkage disequilibrium. Mol. Biol. Evol..

[56] Song K., Li L., Zhang G. (2016). Coverage recommendation for genotyping analysis of highly heterologous species using next-generation sequencing technology. Sci. Rep..

[57] Xie X., Yang Y., Ren Q., Ding X., Bao P., Yan B., Yan X., Han J., Yan P., Qiu Q. (2018). Accumulation of deleterious mutations in the domestic yak genome. Anim. Genet..

[58] Mahar K., Gurao A., Kumar A., Pratap Singh L., Chitkara M., Gowane G.R., Ahlawat S., Niranjan S.K., Pundir R.K., Kataria R.S. (2024). Genomic inbreeding analysis reveals resilience and genetic diversity in Indian yak populations. Gene.

[59] Chen S.-Y., Luo Z., Jia X., Zhou J., Lai S.-J. (2024). Evaluating genomic inbreeding of two Chinese yak (*Bos grunniens*) populations. BMC Genom..

[60] Wang Q., Lan T., Li H., Sahu S.K., Shi M., Zhu Y., Han L., Yang S., Li Q., Zhang L. (2022). Whole-genome resequencing of Chinese pangolins reveals a population structure and provides insights into their conservation. Commun. Biol..

[61] Yang S., Liu Y., Zhao X., Chen J., Li H., Liang H., Fan J., Zhou M., Wang S., Zhang X. (2024). Genomic exploration of the endangered oriental stork, *Ciconia boyciana*, sheds light on migration adaptation and future conservation. GigaScience.

[62] Subramanian S., Kumar M. (2024). The Association between the Abundance of Homozygous Deleterious Variants and the Morbidity of Dog Breeds. Biology.

[63] Jiang Y., Li X., Liu J., Zhang W., Zhou M., Wang J., Liu L., Su S., Zhao F., Chen H. (2022). Genome-wide detection of genetic structure and runs of homozygosity analysis in Anhui indigenous and Western commercial pig breeds using PorcineSNP80k data. BMC Genom..

[64] Dixit S.P., Singh S., Ganguly I., Bhatia A.K., Sharma A., Kumar N.A., Dang A.K., Jayakumar S. (2020). Genome-Wide Runs of Homozygosity Revealed Selection Signatures in *Bos indicus*. Front. Genet..

[65] Charlesworth D., Willis J.H. (2009). The genetics of inbreeding depression. Nat. Rev. Genet..

[66] Cruz F., Vila C., Webster M.T. (2008). The Legacy of Domestication: Accumulation of Deleterious Mutations in the Dog Genome. Mol. Biol. Evol..

[67] Skorczyk A., Flisikowski K., Switonski M. (2012). A comparative analysis of MC4R gene sequence, polymorphism, and chromosomal localization in Chinese raccoon dog and Arctic fox. DNA Cell Biol..

[68] Li Y.M., Si S., Guo P.C., Li L.L., Bai C.Y., Yan S.Q. (2015). Cloning and identification of the ASIP gene in Chinese raccoon dog (*Nyctereutes procyonoides procyonoides*). Genet. Mol. Res. GMR.

[69] Dong Y., Zhang X., Xie M., Arefnezhad B., Wang Z., Wang W., Feng S., Huang G., Guan R., Shen W. (2015). Reference genome of wild goat (*Capra aegagrus*) and sequencing of goat breeds provide insight into genic basis of goat domestication. BMC Genom..

[70] Moore R.K., Shimasaki S. (2005). Molecular biology and physiological role of the oocyte factor, BMP-15. Mol. Cell. Endocrinol..

[71] Bhat B., Singh A., Iqbal Z., Kaushik J.K., Rao A.R., Ahmad S.M., Bhat H., Ayaz A., Sheikh F.D., Kalra S. (2019). Comparative transcriptome analysis reveals the genetic basis of coat color variation in Pashmina goat. Sci. Rep..

[72] Zhang B., Chang L., Lan X., Asif N., Guan F., Fu D., Li B., Yan C., Zhang H., Zhang X. (2018). Genome-wide definition of selective sweeps reveals molecular evidence of trait-driven domestication among elite goat (*Capra* species) breeds for the production of dairy, cashmere, and meat. GigaScience.

[73] Wang X., Liu J., Zhou G., Guo J., Yan H., Niu Y., Li Y., Yuan C., Geng R., Lan X. (2016). Whole-genome sequencing of eight goat populations for the detection of selection signatures underlying production and adaptive traits. Sci. Rep..

[74] Hubbard J.K., Uy J.A.C., Hauber M.E., Hoekstra H.E., Safran R.J. (2010). Vertebrate pigmentation: From underlying genes to adaptive function. Trends Genet..

[75] Benjelloun B., Alberto F.J., Streeter I., Boyer F., Coissac E., Stucki S., BenBati M., Ibnelbachyr M., Chentouf M., Bechchari A. (2015). Characterizing neutral genomic diversity and selection signatures in indigenous populations of Moroccan goats (*Capra hircus*) using WGS data. Front. Genet..

[76] Woodcock M.R., Vaughn-Wolfe J., Elias A., Kump D.K., Kendall K.D., Timoshevskaya N., Timoshevskiy V., Perry D.W., Smith J.J., Spiewak J.E. (2017). Identification of Mutant Genes and Introgressed Tiger Salamander DNA in the Laboratory Axolotl, *Ambystoma mexicanum*. Sci. Rep..

[77] Square T.A., Jandzik D., Massey J.L., Romášek M., Stein H.P., Hansen A.W., Purkayastha A., Cattell M.V., Medeiros D.M. (2020). Evolution of the endothelin pathway drove neural crest cell diversification. Nature.

[78] Guo J., Tao H., Li P., Li L., Zhong T., Wang L., Ma J., Chen X., Song T., Zhang H. (2018). Whole-genome sequencing reveals selection signatures associated with important traits in six goat breeds. Sci. Rep..

[79] Praetorius C., Grill C., Stacey S.N., Metcalf A.M., Gorkin D.U., Robinson K.C., Van Otterloo E., Kim R.S., Bergsteinsdottir K., Ogmundsdottir M.H. (2013). A Polymorphism in IRF4 Affects Human Pigmentation through a Tyrosinase-Dependent MITF/TFAP2A Pathway. Cell.

[80] Han J., Kraft P., Nan H., Guo Q., Chen C., Qureshi A., Hankinson S.E., Hu F.B., Duffy D.L., Zhao Z.Z. (2008). A genome-wide association study identifies novel alleles associated with hair color and skin pigmentation. PLoS Genet..

[81] Sulem P., Gudbjartsson D.F., Stacey S.N., Helgason A., Rafnar T., Magnusson K.P., Manolescu A., Karason A., Palsson A., Thorleifsson G. (2007). Genetic determinants of hair, eye and skin pigmentation in Europeans. Nat. Genet..

[82] Nan H., Kraft P., Qureshi A.A., Guo Q., Chen C., Hankinson S.E., Hu F.B., Thomas G., Hoover R.N., Chanock S. (2009). Genome-wide association study of tanning phenotype in a population of European ancestry. J. Investig. Dermatol..

[83] Chhotaray S., Panigrahi M., Bhushan B., Gaur G., Dutt T., Mishra B., Singh R. (2021). Genome-wide association study reveals genes crucial for coat color production in Vrindavani cattle. Livest. Sci..

[84] Hu S., Chen Y., Zhao B., Yang N., Chen S., Shen J., Bao G., Wu X. (2020). KIT is involved in melanocyte proliferation, apoptosis and melanogenesis in the Rex Rabbit. PeerJ.

[85] Zhang C., Xu M., Yang M., Liao A., Lv P., Liu X., Chen Y., Liu H., He Z. (2024). Efficient generation of cloned cats with altered coat colour by editing of the KIT gene. Theriogenology.

[86] Kottler V.A., Fadeev A., Weigel D., Dreyer C. (2013). Pigment pattern formation in the guppy, *Poecilia reticulata*, involves the Kita and Csf1ra receptor tyrosine kinases. Genetics.

[87] Otsuki Y., Okuda Y., Naruse K., Saya H. (2020). Identification of kit-ligand a as the Gene Responsible for the Medaka Pigment Cell Mutant few melanophore. G3.

[88] Picardo M., Cardinali G. (2011). The genetic determination of skin pigmentation: KITLG and the KITLG/c-Kit pathway as key players in the onset of human familial pigmentary diseases. J. Investig. Dermatol..

[89] Wang J., Li W., Zhou N., Liu J., Zhang S., Li X., Li Z., Yang Z., Sun M., Li M. (2021). Identification of a novel mutation in the KITLG gene in a Chinese family with familial progressive hyper- and hypopigmentation. BMC Med. Genom..

[90] Horrell E.M.W., Boulanger M.C., D’orazio J.A. (2016). Melanocortin 1 receptor: Structure, function, and regulation. Front. Genet..

[91] Nasti T.H., Timares L. (2014). MC1R, Eumelanin and Pheomelanin: Their role in determining the susceptibility to skin cancer. Photochem. Photobiol..

[92] Wei C.-Y., Zhu M.-X., Lu N.-H., Peng R., Yang X., Zhang P.-F., Wang L., Gu J.-Y. (2019). Bioinformatics-based analysis reveals elevated MFSD12 as a key promoter of cell proliferation and a potential therapeutic target in melanoma. Oncogene.

[93] Del Bino S., Duval C., Bernerd F. (2018). Clinical and biological characterization of skin pigmentation diversity and its consequences on UV impact. Int. J. Mol. Sci..

[94] Kawakami A., Fisher D.E. (2017). The master role of microphthalmia-associated transcription factor in melanocyte and mela-noma biology. Lab. Investig..

[95] Wang C., Kocher T.D., Wu J., Li P., Liang G., Lu B., Xu J., Chen X., Wang D. (2023). Knockout of microphthalmia-associated transcription factor (mitf) confers a red and yellow tilapia with few pigmented melanophores. Aquaculture.

[96] Kim D.-H., Lee J., Ko J.-K., Lee K. (2024). Melanophilin regulates dendritogenesis in melanocytes for feather pigmentation. Commun. Biol..

[97] Yuan Z., Zhang X., Pang Y., Qi Y., Wang Q., Hu Y., Zhao Y., Ren S., Huo L. (2023). Association analysis of melanophilin (*MLPH*) gene expression and polymorphism with plumage color in quail. Arch. Anim. Breed..

[98] Zhang H., Wu Z., Yang L., Zhang Z., Chen H., Ren J. (2021). Novel mutations in the *Myo5a* gene cause a dilute coat color phenotype in mice. FASEB J..

[99] O’Sullivan T.N., Wu X.S., Rachel R.A., Huang J.-D., Swing D.A., Matesic L.E., Hammer J.A., Copeland N.G., Jenkins N.A. (2004). Dsu functions in a MYO5A-independent pathway to suppress the coat color of dilute mice. Proc. Natl. Acad. Sci. USA.

[100] Kidd K.K., Pakstis A.J., Donnelly M.P., Bulbul O., Cherni L., Gurkan C., Kang L., Li H., Yun L., Paschou P. (2020). The distinctive geographic patterns of common pigmentation variants at the OCA2 gene. Sci. Rep..

[101] Vacher J., Bruccoleri M., Pata M. (2020). Ostm1 from mouse to human: Insights into osteoclast maturation. Int. J. Mol. Sci..

[102] Pandruvada S.N.M., Beauregard J., Benjannet S., Pata M., Lazure C., Seidah N.G., Vacher J. (2016). Role of Ostm1 cytosolic complex with kinesin 5B in intracellular dispersion and trafficking. Mol. Cell. Biol..

[103] Watt B., van Niel G., Raposo G., Marks M.S. (2013). PMEL: A pigment cell-specific model for functional amyloid formation. Pigment Cell Melanoma Res..

[104] Hee J.S., Mitchell S.M., Liu X., Leonhardt R.M. (2017). Melanosomal formation of PMEL core amyloid is driven by aromatic residues. Sci. Rep..

[105] Yoshida-Amano Y., Hachiya A., Ohuchi A., Kobinger G.P., Kitahara T., Takema Y., Fukuda M. (2012). Essential role of RAB27A in determining constitutive human skin color. PLoS ONE.

[106] Jacobs L.C., Hamer M.A., Gunn D.A., Deelen J., Lall J.S., van Heemst D., Uh H.-W., Hofman A., Uitterlinden A.G., Griffiths C.E. (2015). A genome-wide association study identifies the skin color genes IRF4, MC1R, ASIP, and BNC2 influencing facial pigmented spots. J. Investig. Dermatol..

[107] Liu F., Visser M., Duffy D.L., Hysi P.G., Jacobs L.C., Lao O., Zhong K., Walsh S., Chaitanya L., Wollstein A. (2015). Genetics of skin color variation in Europeans: Genome-wide association studies with functional follow-up. Hum. Genet..

[108] Twumasi G., Wang H., Xi Y., Qi J., Li L., Bai L., Liu H. (2023). Genome-Wide Association Studies Reveal Candidate Genes Associated with Pigmentation Patterns of Single Feathers of Tianfu Nonghua Ducks. Animals.

[109] Al Mahi A., Ablain J. (2022). RAS pathway regulation in melanoma. Dis. Model. Mech..

[110] Costin G.-E., Hearing V.J. (2007). Human skin pigmentation: Melanocytes modulate skin color in response to stress. FASEB J..

[111] Naik P.P., Farrukh S.N. (2022). Influence of ethnicities and skin color variations in different populations: A review. Skin Pharmacol. Physiol..

[112] Batai K., Cui Z., Arora A., Shah-Williams E., Hernandez W., Ruden M., Hollowell C.M.P., Hooker S.E., Bathina M., Murphy A.B. (2021). Genetic loci associated with skin pigmentation in African Americans and their effects on vitamin D deficiency. PLoS Genet..

[113] Branicki W., Brudnik U., Draus-Barini J., Kupiec T., Wojas-Pelc A. (2008). Association of the SLC45A2 gene with physio-logical human hair colour variation. J. Hum. Genet..

[114] Huo L., Zhang X., Pang Y., Qi Y., Ren S., Wu F., Shang Y., Xi J. (2024). Expression and Mutation of SLC45A2 Affects Iris Color in Quail. J. Poult. Sci..

[115] Wang L.-M., Bu H.-Y., Song F.-B., Zhu W.-B., Fu J.-J., Dong Z.-J. (2019). Characterization and functional analysis of slc7a11 gene, involved in skin color differentiation in the red tilapia. Comp. Biochem. Physiol. Part A Mol. Integr. Physiol..

[116] Chen Y., Hu S., Mu L., Zhao B., Wang M., Yang N., Bao G., Zhu C., Wu X. (2019). *Slc7a11* Modulated by *POU2F1* is Involved in Pigmentation in Rabbit. Int. J. Mol. Sci..

[117] Arnoldi A., Tonelli A., Crippa F., Villani G., Pacelli C., Sironi M., Pozzoli U., D’Angelo M.G., Meola G., Martinuzzi A. (2008). A clinical, genetic, and biochemical characterization of SPG7 mutations in a large cohort of patients with hereditary spastic paraplegia. Hum. Mutat..

[118] Yee N.S., Kazi A.A., Yee R.K. (2014). Cellular and developmental biology of TRPM7 channel-kinase: Implicated roles in cancer. Cells.

[119] Shchagina O., Stepanova A., Mishakova P., Kadyshev V., Demina N., Bessonova L., Ionova S., Guseva D., Marakhonov A., Zinchenko R. (2024). Common Variants in the *TYR* Gene with Unclear Pathogenicity as the Cause of Oculocutaneous Albinism in a Cohort of Russian Patients. Biomedicines.

[120] Lai X., Wichers H.J., Soler-Lopez M., Dijkstra B.W. (2017). Structure and function of human tyrosinase and tyrosinase-related proteins. Chem.–A Eur. J..

[121] Wagatsuma T., Suzuki E., Shiotsu M., Sogo A., Nishito Y., Ando H., Hashimoto H., Petris M.J., Kinoshita M., Kambe T. (2023). Pigmentation and TYRP1 expression are mediated by zinc through the early secretory pathway-resident ZNT proteins. Commun. Biol..

[122] Gissen P., Johnson C.A., Gentle D., Hurst L.D., Doherty A.J., O’Kane C.J., Kelly D.A., Maher E.R. (2005). Comparative evolutionary analysis of VPS33 homologues: Genetic and functional insights. Hum. Mol. Genet..

[123] Graham S.C., Wartosch L., Gray S.R., Scourfield E.J., Deane J.E., Luzio J.P., Owen D.J. (2013). Structural basis of Vps33A recruitment to the human HOPS complex by Vps16. Proc. Natl. Acad. Sci. USA.

[124] Harney É., Patterson N., Reich D., Wakeley J. (2021). Assessing the performance of qpAdm: A statistical tool for studying popu-lation admixture. Genetics.

[125] Zhang C., Dong S.-S., Xu J.-Y., He W.-M., Yang T.-L. (2019). PopLDdecay: A fast and effective tool for linkage disequilibrium decay analysis based on variant call format files. Bioinformatics.

