# Supplementary material for: Population Genomics Reveals Elevated Inbreeding and Accumulation of Deleterious Mutations in White Raccoon Dogs
Source: Biology (Basel). 2025 Jan 2;14(1):30. doi: 10.3390/biology14010030 (PMC11760849; doi:10.3390/biology14010030)
Supplement: Supplementary file 1 [file biology-14-00030-s001.zip › biology-3367151-supplementary.pdf]

Supplemental material

Supplemental Figure S

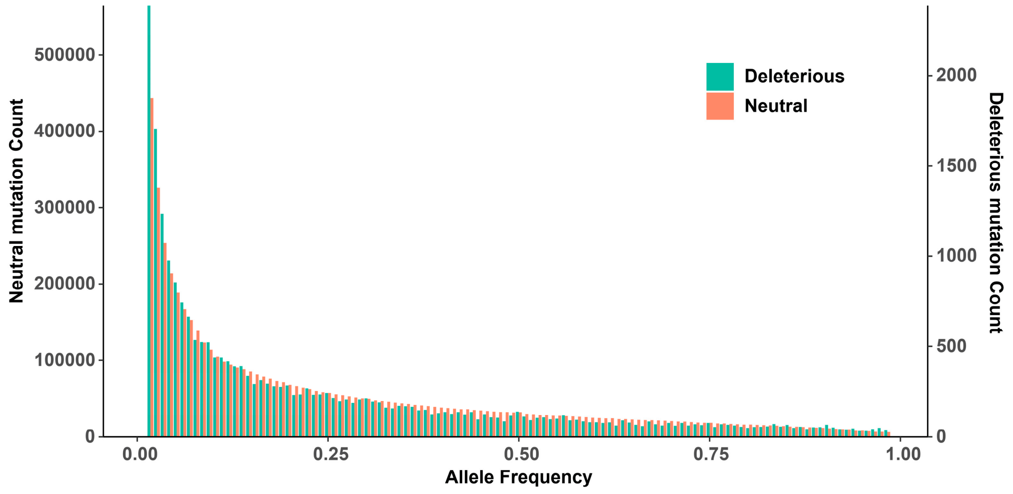

Figure S1. Site-frequency spectrum for deleterious variants (Missenses, LoF and dnsSNP mutations combined) and neutral variants (Intergenic mutations) for the raccoon dog populations. The number of loci (y axis) is shown for each allele frequency (x axis).

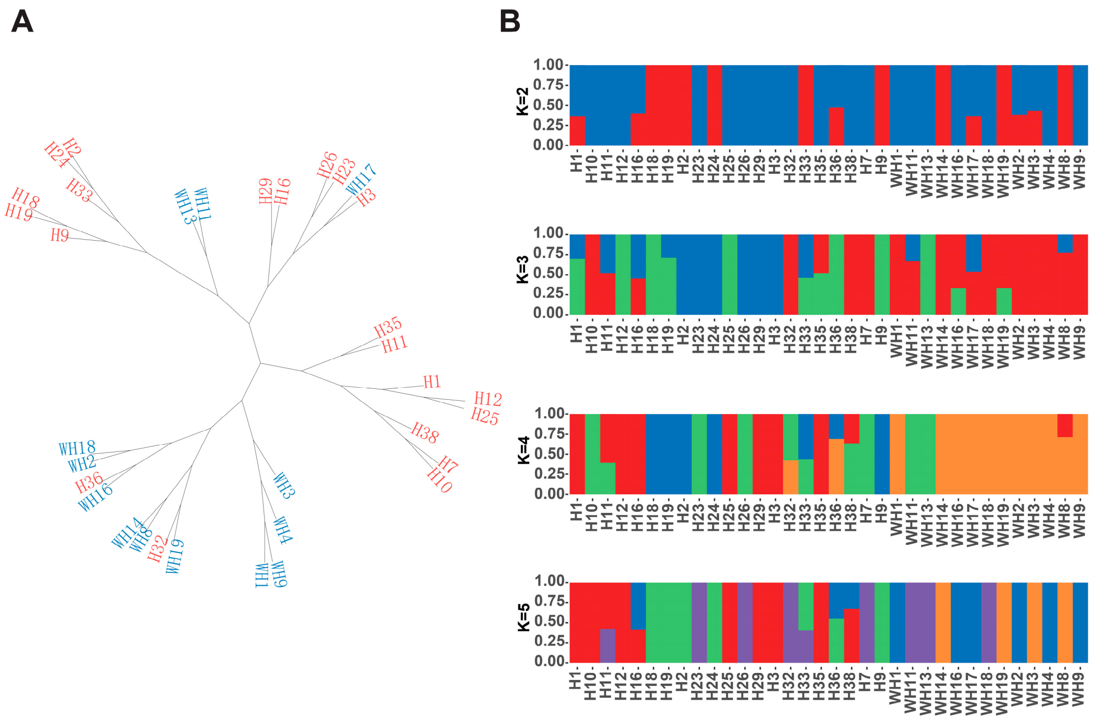

Figure S2. (A) Maximum-likelihood phylogenetic tree of raccoon dog populations and (B) Admixture analysis results based on K from 2 to 5 for the raccoon dog population.

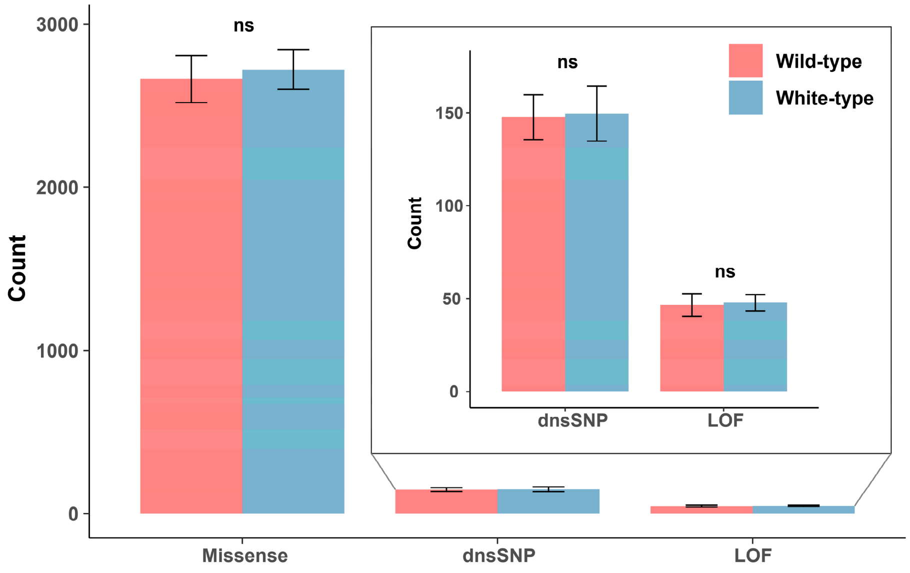

Figure S3. The number of homozygous genotypes of mutational load between the white-type raccoon dogs and wild-type raccoon dogs.

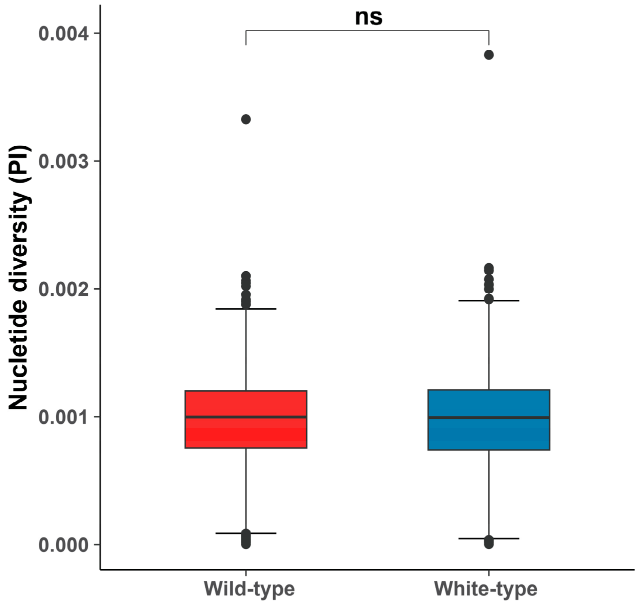

Figure S4. The genetic diversity between wild-type and white-type raccoon dogs.

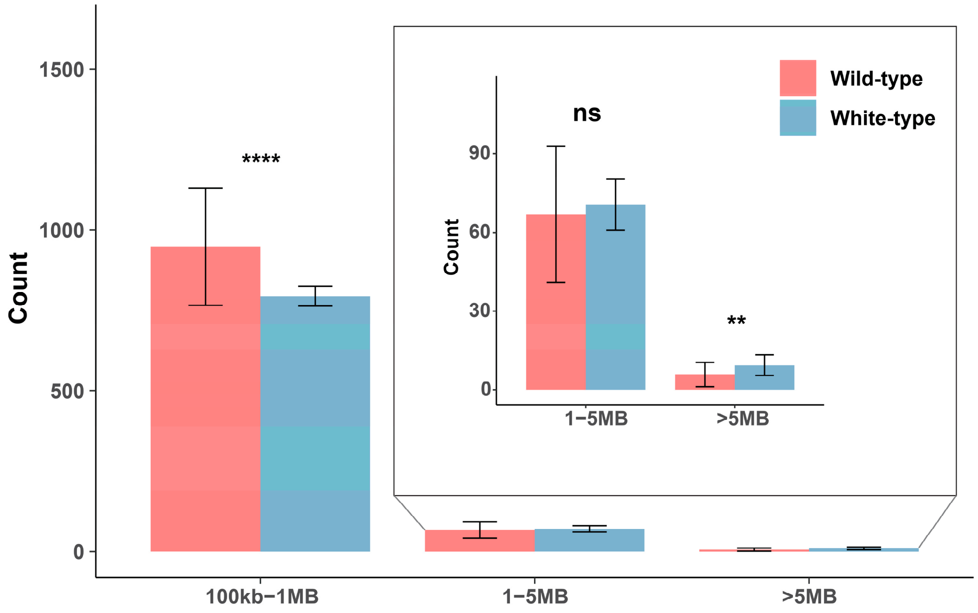

Figure S5. The number of homozygous genotypes of ROH length between the white-type raccoon dogs and wild-type raccoon dogs.

Supplemental Tables

Table S1. Summary of Software Used in the Study.

Software	Version	Source
Trimmomatic	0.33.0	[1]
BWA	0.7.10-r789	[2]
Picard tools	2.1.1	http://picard.sourceforge.net
GATK	4.0.3.0	[3]
VCFtools	0.1.16	https://github.com/vcftools/vcftools/releases/tag/v0.1.16
KING	2.2.7	https://www.kingrelatedness.com/
ANNOVAR	2015-12-14	[4]
GCTA	1.91.4beta3	[5]
vcf2phylip	2.7	[6]
IQ-TREE	2.3.6	[7]
ADMIXTURE	1.3.0	[8]
PopLDdecay	3.43	[9]
PLINK	1.90b6.10	[10]
SnpEff	v.5.0e	[11]
MSMC2	v2.1.4	[12]
BEAGLE	5.0	[13]
GONE	No releases published	https://github.com/esrud/GONE

Table S2. Results of Kinship coefficients and propIBD estimation with the KING program.

ID1	ID2	Kinship	PropIBD	InfType
H10	H15	0.1285	0.1968	2nd
H10	H17	0.091	0.1379	3rd
H10	H20	0.0678	0.1177	3rd
H11	H14	0.117	0.1197	3rd
H11	H22	0.197	0.2981	2nd
H14	H22	0.1279	0.1119	3rd
H15	H17	0.0949	0.1343	3rd
H15	H20	0.0826	0.1205	3rd
H16	H21	0.2084	0.3241	FS
H17	H20	0.2203	0.2774	2nd
H10	H37	0.1018	0.2142	2nd
H10	H39	0.1172	0.2012	2nd
H15	H37	0.0831	0.1774	2nd
H15	H39	0.1229	0.1689	3rd
H16	H34	0.2328	0.2186	2nd
H17	H37	0.264	0.4491	FS
H17	H39	0.1011	0.1447	3rd
H20	H37	0.2224	0.3572	FS
H20	H39	0.1371	0.1929	2nd
H21	H34	0.2303	0.2384	2nd
H23	H27	0.1709	0.1728	3rd
H10	H6	0.1264	0.1719	3rd
H10	H8	0.1219	0.2087	2nd
H13	H4	0.221	0.3463	FS
H15	H6	0.0803	0.1229	3rd
H15	H8	0.1144	0.1761	3rd
H17	H5	0.0104	0.0889	3rd
H17	H6	0.1058	0.1465	3rd
H17	H8	0.2515	0.4387	FS
H20	H6	0.1045	0.1028	3rd
H20	H8	0.2441	0.385	FS
H24	H31	0.0837	0.1024	3rd
H29	H30	0.2393	0.4402	FS
H32	H37	0.0012	0.0919	3rd
H37	H39	0.0894	0.1845	2nd
H37	H6	0.075	0.1266	3rd
H37	H8	0.2674	0.4983	FS
H39	H6	0.116	0.1574	3rd
H39	H8	0.1436	0.2311	2nd
H37	WH20	0.0245	0.0936	3rd
H5	H8	0.0079	0.1014	3rd
H6	H8	0.1391	0.1732	3rd

WH11	WH12	0.1168	0.1691	3rd
WH13	WH15	0.2688	0.4507	FS
WH2	WH7	0.1266	0.1013	3rd
WH20	WH21	0.0896	0.2093	2nd
WH4	WH5	0.2021	0.2386	2nd
WH4	WH6	0.1807	0.2626	2nd
WH5	WH6	0.2284	0.3455	FS

Note:

ID1: Individual ID for the first individual of the pair

ID2: Individual ID for the second individual of the pair

Kinship: Estimated kinship coefficient (φ) from the SNP data

PropIBD: Proportion of genomes shared identical-by-descent, estimated by IBD2Seg + IBD1Seg/2, estimate of $\pi=\pi2+\pi1/2$

InfType: Inferred relationship type, FS, 2rd, and 3rd means full-siblings, 2nd-degree, and 3rd-degree relationships respectively.

Table S3. Information and statistics of the whole genome resequencing data of 58 raccoon dogs.

Sample ID	Color Type	Mapping Rate	Sequencing Depth (×)	Sequencing Coverage	Duplication Rate	Gender
H10	Wild-type	99.77%	14.51	98.32%	1.97%	Male
H11	Wild-type	99.77%	13.72	98.27%	2.11%	Male
H12	Wild-type	99.68%	13	97.95%	1.95%	Male
H13	Wild-type	99.64%	12.63	97.90%	2.75%	Male
H14	Wild-type	99.74%	12.04	97.69%	1.56%	Male
H15	Wild-type	99.79%	13.04	98.20%	1.64%	Male
H16	Wild-type	99.78%	13.67	98.32%	1.21%	Male
H17	Wild-type	99.79%	13.35	98.00%	2.06%	Male
H18	Wild-type	99.68%	13.22	97.97%	2%	Male
H19	Wild-type	99.72%	13.2	97.89%	1.73%	Male
H1	Wild-type	99.77%	12.7	97.70%	1.35%	Male
H20	Wild-type	99.51%	12.71	97.80%	1.78%	Male
H21	Wild-type	99.78%	13.44	98.09%	1.73%	Male
H22	Wild-type	99.73%	13.07	98.19%	1.40%	Male
H23	Wild-type	99.66%	12.65	98.29%	1.30%	Female
H24	Wild-type	99.65%	10.95	96.66%	5.07%	Male
H25	Wild-type	99.75%	13.62	98.22%	1.61%	Male
H26	Wild-type	99.77%	13.66	98.37%	1.83%	Male
H27	Wild-type	99.70%	12.82	97.98%	2.13%	Male
H29	Wild-type	99.80%	15.08	97.89%	1.41%	Male
H2	Wild-type	99.77%	14.59	98.21%	1.02%	Male
H30	Wild-type	99.76%	15	98.87%	1.20%	Female
H31	Wild-type	99.76%	15.18	97.99%	1.37%	Male
H32	Wild-type	99.74%	15.06	97.75%	2.18%	Male
H33	Wild-type	99.73%	15.23	97.57%	1.21%	Male
H34	Wild-type	99.76%	12.54	98.26%	1.02%	Female
H35	Wild-type	99.75%	12.6	98.19%	2.18%	Female
H36	Wild-type	99.84%	16.23	98.20%	1.10%	Male
H37	Wild-type	99.84%	14.68	98.75%	0.80%	Male
H38	Wild-type	99.82%	11.96	97.59%	0.99%	Male
H39	Wild-type	99.81%	13.62	98.27%	0.76%	Male
H3	Wild-type	99.78%	12.06	98.19%	1.88%	Female
H4	Wild-type	99.69%	14.83	98.58%	2.34%	Male
H5	Wild-type	99.80%	14.82	98.53%	1.12%	Male
H6	Wild-type	99.77%	13.24	98.08%	1.75%	Male
H7	Wild-type	99.77%	11.34	97.18%	1.74%	Male
H8	Wild-type	99.81%	14.15	98.47%	1.21%	Male
H9	Wild-type	99.77%	13.56	98.16%	1.39%	Male
WH11	White-type	99.89%	13.14	97.73%	0.76%	Male
WH12	White-type	99.87%	14.54	97.93%	0.97%	Male
WH13	White-type	99.89%	13.87	97.75%	0.67%	Male

WH14	White-type	99.89%	13.8	97.73%	0.67%	Male
WH15	White-type	99.88%	14.02	97.82%	0.74%	Male
WH16	White-type	99.89%	13.11	97.69%	0.54%	Male
WH17	White-type	99.87%	12.97	97.52%	0.62%	Male
WH18	White-type	99.88%	14.97	97.87%	0.59%	Male
WH19	White-type	99.88%	13.86	97.80%	0.64%	Male
WH1	White-type	99.85%	13.07	98.06%	0.82%	Male
WH20	White-type	99.86%	18.37	98.07%	0.56%	Male
WH21	White-type	99.88%	12.12	97.19%	0.52%	Male
WH2	White-type	99.86%	14.01	98.30%	1.08%	Male
WH3	White-type	99.86%	17.26	98.01%	0.79%	Male
WH4	White-type	99.86%	13.2	97.75%	0.75%	Male
WH5	White-type	99.88%	13.4	97.93%	0.69%	Male
WH6	White-type	99.89%	13.08	97.82%	0.69%	Male
WH7	White-type	99.88%	12.33	97.70%	0.72%	Male
WH8	White-type	99.88%	13.82	97.70%	0.65%	Male
WH9	White-type	99.87%	12.88	97.63%	0.62%	Male

Table S4. Summary of the SNP sites.

Variant type	SNP Count
Intron	2291755
Intergenic	5697884
Splicing	217
exonic	56923
Nonsynonymous	23334
Synonymous	33274
stopgain	303
stoploss	12

Table S5. Summary of functions of color-related genes.

Gene	Function	Reference
ADAMTS20	Associate with coloration of the coat	[14-16]
AHCY	Relate to skin color in elite goats	[17]
ASIP	Encode agouti signalling proteins that promote hair follicle melanocytes to synthesize pheomelanin in animals	[17-19]
ATRN	Associate with coloration of the coat	[14-16]
ATRN1	Associate with coloration of the coat	[14-16]
ATRNL1	Associate with coloration of the coat	[14-16]
DST	Determination of black coat color in goats	[18,20]
EDN3	A crucial role in the endocytosis of melanocytes	[21]
EDNRB	A crucial role in regulating melanocyte development and function, thereby influencing pigmentation in skin and hair.	[22]
EIF2S2	Influence the skin and hair pigmentation	[19,23]
EXOC2	Associate with skin pigmentation, hair color, or skin sensitivity	[23-27]
GNA11	Determination of black coat color in goats	[18,20]
GNAQ	Associate with coloration of the coat	[14-16]
HELLS	Associate with coloration of the coat	[14-16]
HTT	Determination of black coat color in goats	[18,20]
IRF4	Associate with skin pigmentation, hair color, or skin sensitivity	[23-27]

KIT	The encoded protein is the receptor tyrosine kinase for stem cell factor (SCF), which plays a key role in the migration and differentiation of melanocytes.	[28-30]
KITA	The development and function of melanocytes and xanthophore cells.	[31,32]
KITLG	A pivotal role in pigmentation by encoding the ligand for the receptor tyrosine kinase KIT	[33,34]
MC1R	Regulate skin pigmentation, UV responses, and melanoma risk, as it controls the production of eumelanin (black/brown) and pheomelanin (red/yellow), determining the amount and type of melanin synthesized by melanocytes	[35,36]
MFSD12	Associate with skin pigmentation as it encodes a transporter that mediates the import of cysteine into melanosomes, regulating skin pigmentation by affecting the production of cysteinyldopas used in pheomelanin synthesis	[37,38]
MITF	A critical role in color-related functions by regulating the development, survival, and pigmentation of melanocytes, which produce melanin responsible for skin, hair, and eye color.	[39,40]
MLPH	It is crucial for melanosome transport and dendritogenesis in melanocytes, which are essential for feather and skin pigmentation	[41,42]

MYO5A	Essential for melanosome transport in melanocytes, which is critical for the distribution of melanin and thus affects skin, hair, and eye color	[43,44]
OCA2	Associate with pigmentation, playing a key role in the production of melanin, which determines eye, hair, and skin color	[27,45]
OSTM1	Associate with osteoclast function and potentially with pigmentation	[46,47]
PMEL	Crucial for melanosome morphogenesis and pigmentation, encoding a protein that forms amyloid fibrils on which melanin is deposited	[48,49]
RAB27A	Essential for melanosome transport in melanocytes, which is critical for skin, hair, and eye pigmentation	[50]
RALY	Relevant to pigmentation, particularly in the context of embryonic lethality of homozygous lethal yellow mice, suggesting a potential role in melanogenesis.	[51,52]
RALYL	Involve in regulating pigmentation and color patterns.	[53]
RASGEF1B	Involve in Ras signaling, which may indirectly influence pigmentation by affecting melanocyte function.	[54,55]
SLC24A5	A key role in pigmentation by regulating melanin production in melanocytes	[56,57]
SLC45A2	Influence pigmentation, affecting hair, skin, and eye color	[58,59]

SLC7A11	Crucial for pheomelanin synthesis, affecting coloration in mammals	[60,61]
SPG7	Indirectly affect the metabolism and distribution of cellular pigments	[62]
TRPM7	Influence color-related cellular functions through ion transport.	[63]
TYR	Encode the enzyme tyrosinase, which is essential for melanin production and determines skin, hair, and eye color	[64]
TYRO	Play a critical role in melanin synthesis, influencing pigmentation traits such as skin, hair, and eye color.	[65,66]
TYRP1	Involve in the synthesis of eumelanin, a key pigment in determining human skin, hair, and eye color	[65,66]
VPS33A	Involve in the transport of pigment granules, which affects coloration in organisms such as Drosophila melanogaster	[67,68]

Table S6. A summary of deleterious mutations in color-related genes region.

Chromosome	Position	Type	Gene	Allele Frequency Difference
chr13	54111342	missense	AHCY	0.059211
chr13	54111353	missense	AHCY	0.072368
chr13	54111551	missense	AHCY	0.198684
chr13	54111617	missense	AHCY	0.072368
chr13	54111863	missense	AHCY	0.185526
chr13	54111955	missense	AHCY	0.072368
chr13	54111965	missense	AHCY	0.072368
chr13	54112036	missense	AHCY	0.267105
chr13	54112278	missense	AHCY	-0.01579
chr13	54112308	missense	AHCY	0.313158
chr13	54112379	missense	AHCY	0.313158
chr22	21413421	missense	AHCY	-0.00132
chr4	96416921	missense	AHCY	-0.06579
chr1	88425575	missense	RALY	-0.13289
chr1	88425575	dnsSNP	RALY	-0.13289
chr2	88736626	missense	TRPM7	-0.03947
chr6	35395811	missense	TRPM7	-0.11184
chr6	38992558	missense	TRPM7	-0.07237
chr6	39026775	missense	TRPM7	0.010526
chr6	39057800	missense	TRPM7	-0.02632
chr5	59574389	missense	OCA2	-0.06711
chr5	59575103	missense	OCA2	0.103947
chr5	59652531	missense	OCA2	0.036842
chr5	59809908	missense	OCA2	-0.12105
chr5	59832693	missense	OCA2	0.110526

Note:

Allele Frequency Difference means the Allele Frequency of the white-type raccoon dogs minus the Allele Frequency of the wild-type raccoon dogs

Table S7. Summary of functions of significantly differential color-related genes.

Gene	Function	Reference
AHCY	Related to skin color in elite goats	[17]
OCA2	Associated with pigmentation, playing a key role in the production of melanin, which determines eye, hair, and skin color	[27,45]
RALY	Influence color-related cellular functions through ion transport.	[51,52]
TRPM7	Influence color-related cellular functions through ion transport.	[63]

Reference

1. Bolger, A.M.; Lohse, M.; Usadel, B.J.B. Trimmomatic: a flexible trimmer for Illumina sequence data. **2014**, *30*, 2114-2120.
2. Li, H.J.a.p.a. Aligning sequence reads, clone sequences and assembly contigs with BWA-MEM. **2013**.
3. McKenna, A.; Hanna, M.; Banks, E.; Sivachenko, A.; Cibulskis, K.; Kernytsky, A.; Garimella, K.; Altshuler, D.; Gabriel, S.; Daly, M.J.G.r. The Genome Analysis Toolkit: a MapReduce framework for analyzing next-generation DNA sequencing data. **2010**, *20*, 1297-1303.
4. Wang, K.; Li, M.; Hakonarson, H.J.N.a.r. ANNOVAR: functional annotation of genetic variants from high-throughput sequencing data. **2010**, *38*, e164-e164.
5. Yang, J.; Lee, S.H.; Goddard, M.E.; Visscher, P.M.J.T.A.J.o.H.G. GCTA: a tool for genome-wide complex trait analysis. **2011**, *88*, 76-82.
6. Ortiz, E.M. vcf2phylip v2. 0: convert a VCF matrix into several matrix formats for phylogenetic analysis. **2019**.
7. Nguyen, L.-T.; Schmidt, H.A.; Von Haeseler, A.; Minh, B.Q.J.M.b.; evolution. IQ-

TREE: a fast and effective stochastic algorithm for estimating maximum-likelihood phylogenies. **2015**, *32*, 268-274.
8. Harney, É.; Patterson, N.; Reich, D.; Wakeley, J.J.G. Assessing the performance of qpAdm: a statistical tool for studying population admixture. **2021**, *217*, iyaa045.
9. Zhang, C.; Dong, S.-S.; Xu, J.-Y.; He, W.-M.; Yang, T.-L.J.B. PopLDdecay: a fast and effective tool for linkage disequilibrium decay analysis based on variant call format files. **2019**, *35*, 1786-1788.
10. Purcell, S.; Neale, B.; Todd-Brown, K.; Thomas, L.; Ferreira, M.A.; Bender, D.; Maller, J.; Sklar, P.; De Bakker, P.I.; Daly, M.J.J.T.A.j.o.h.g. PLINK: a tool set for whole-genome association and population-based linkage analyses. **2007**, *81*, 559-575.
11. Cingolani, P.; Platts, A.; Wang, L.L.; Coon, M.; Nguyen, T.; Wang, L.; Land, S.J.; Lu, X.; Ruden, D.M.J.f. A program for annotating and predicting the effects of single nucleotide polymorphisms, SnpEff: SNPs in the genome of Drosophila melanogaster strain w1118; iso-2; iso-3. **2012**, *6*, 80-92.
12. Schiffels, S.; Durbin, R.J.N.g. Inferring human population size and separation history from multiple genome sequences. **2014**, *46*, 919-925.
13. Browning, B.L.; Zhou, Y.; Browning, S.R.J.T.A.J.o.H.G. A one-penny imputed genome from next-generation reference panels. **2018**, *103*, 338-348.
14. Dong, Y.; Zhang, X.; Xie, M.; Arefnezhad, B.; Wang, Z.; Wang, W.; Feng, S.; Huang, G.; Guan, R.; Shen, W.J.B.g. Reference genome of wild goat (capra aegagrus) and sequencing of goat breeds provide insight into genic basis of goat domestication. **2015**, *16*, 1-11.
15. Moore, R.K.; Shimasaki, S.J.M.; endocrinology, c. Molecular biology and physiological role of the oocyte factor, BMP-15. **2005**, *234*, 67-73.
16. Bhat, B.; Singh, A.; Iqbal, Z.; Kaushik, J.K.; Rao, A.; Ahmad, S.M.; Bhat, H.; Ayaz, A.; Sheikh, F.; Kalra, S.J.S.R. Comparative transcriptome analysis reveals the genetic basis of coat color variation in Pashmina goat. **2019**, *9*, 6361.
17. Zhang, B.; Chang, L.; Lan, X.; Asif, N.; Guan, F.; Fu, D.; Li, B.; Yan, C.; Zhang, H.; Zhang, X.J.G. Genome-wide definition of selective sweeps reveals molecular evidence of trait-driven domestication among elite goat (Capra species) breeds for the production of dairy, cashmere, and meat. **2018**, *7*, giy105.
18. Wang, X.; Liu, J.; Zhou, G.; Guo, J.; Yan, H.; Niu, Y.; Li, Y.; Yuan, C.; Geng, R.; Lan, X.J.S.r. Whole-genome sequencing of eight goat populations for the detection of selection signatures underlying production and adaptive traits. **2016**, *6*, 38932.
19. Hubbard, J.K.; Uy, J.A.C.; Hauber, M.E.; Hoekstra, H.E.; Safran, R.J.J.T.i.G. Vertebrate pigmentation: from underlying genes to adaptive function. **2010**, *26*, 231-239.
20. Benjelloun, B.; Alberto, F.J.; Streeter, I.; Boyer, F.; Coissac, E.; Stucki, S.; BenBati, M.; Ibnelbachyr, M.; Chentouf, M.; Bechchari, A.J.F.i.g. Characterizing neutral genomic diversity and selection signatures in indigenous populations of Moroccan goats (Capra hircus) using WGS data. **2015**, *6*, 107.
21. Woodcock, M.R.; Vaughn-Wolfe, J.; Elias, A.; Kump, D.K.; Kendall, K.D.;

Timoshevskaya, N.; Timoshevskiy, V.; Perry, D.W.; Smith, J.J.; Spiewak, J.E.J.S.r. Identification of mutant genes and introgressed tiger salamander DNA in the laboratory axolotl, Ambystoma mexicanum. **2017**, *7*, 6.
22. Square, T.A.; Jandzik, D.; Massey, J.L.; Romášek, M.; Stein, H.P.; Hansen, A.W.; Purkayastha, A.; Cattell, M.V.; Medeiros, D.M.J.N. Evolution of the endothelin pathway drove neural crest cell diversification. **2020**, *585*, 563-568.
23. Guo, J.; Tao, H.; Li, P.; Li, L.; Zhong, T.; Wang, L.; Ma, J.; Chen, X.; Song, T.; Zhang, H.J.S.r. Whole-genome sequencing reveals selection signatures associated with important traits in six goat breeds. **2018**, *8*, 10405.
24. Praetorius, C.; Grill, C.; Stacey, S.N.; Metcalf, A.M.; Gorkin, D.U.; Robinson, K.C.; Van Otterloo, E.; Kim, R.S.; Bergsteinsdottir, K.; Ogmundsdottir, M.H.J.C. A polymorphism in IRF4 affects human pigmentation through a tyrosinase-dependent MITF/TFAP2A pathway. **2013**, *155*, 1022-1033.
25. Han, J.; Kraft, P.; Nan, H.; Guo, Q.; Chen, C.; Qureshi, A.; Hankinson, S.E.; Hu, F.B.; Duffy, D.L.; Zhao, Z.Z.J.P.g. A genome-wide association study identifies novel alleles associated with hair color and skin pigmentation. **2008**, *4*, e1000074.
26. Sulem, P.; Gudbjartsson, D.F.; Stacey, S.N.; Helgason, A.; Rafnar, T.; Magnusson, K.P.; Manolescu, A.; Karason, A.; Palsson, A.; Thorleifsson, G.J.N.g. Genetic determinants of hair, eye and skin pigmentation in Europeans. **2007**, *39*, 1443-1452.
27. Nan, H.; Kraft, P.; Qureshi, A.A.; Guo, Q.; Chen, C.; Hankinson, S.E.; Hu, F.B.; Thomas, G.; Hoover, R.N.; Chanock, S.J.J.o.I.D. Genome-wide association study of tanning phenotype in a population of European ancestry. **2009**, *129*, 2250-2257.
28. Chhotaray, S.; Panigrahi, M.; Bhushan, B.; Gaur, G.; Dutt, T.; Mishra, B.; Singh, R.J.L.S. Genome-wide association study reveals genes crucial for coat color production in Vrindavani cattle. **2021**, *247*, 104476.
29. Hu, S.; Chen, Y.; Zhao, B.; Yang, N.; Chen, S.; Shen, J.; Bao, G.; Wu, X.J.P. KIT is involved in melanocyte proliferation, apoptosis and melanogenesis in the Rex Rabbit. **2020**, *8*, e9402.
30. Zhang, C.; Xu, M.; Yang, M.; Liao, A.; Lv, P.; Liu, X.; Chen, Y.; Liu, H.; He, Z.J.T. Efficient generation of cloned cats with altered coat colour by editing of the KIT gene. **2024**, *222*, 54-65.
31. Kottler, V.A.; Fadeev, A.; Weigel, D.; Dreyer, C.J.G. Pigment pattern formation in the guppy, Poecilia reticulata, involves the Kita and Csf1ra receptor tyrosine kinases. **2013**, *194*, 631-646.
32. Otsuki, Y.; Okuda, Y.; Naruse, K.; Saya, H.J.G.G., Genomes, Genetics. Identification of kit-ligand a as the Gene Responsible for the Medaka Pigment Cell Mutant few melanophore. **2020**, *10*, 311-319.
33. Picardo, M.; Cardinali, G.J.J.o.I.D. The genetic determination of skin pigmentation: KITLG and the KITLG/c-Kit pathway as key players in the onset of human familial pigmentary diseases. **2011**, *131*, 1182-1185.
34. Wang, J.; Li, W.; Zhou, N.; Liu, J.; Zhang, S.; Li, X.; Li, Z.; Yang, Z.; Sun, M.; Li, M.J.B.M.G. Identification of a novel mutation in the KITLG gene in a Chinese

family with familial progressive hyper-and hypopigmentation. **2021**, *14*, 1-7.
35. Wolf Horrell, E.M.; Boulanger, M.C.; D'Orazio, J.A.J.F.i.g. Melanocortin 1 receptor: structure, function, and regulation. **2016**, *7*, 95.
36. Nasti, T.H.; Timares, L.J.P.; photobiology. MC 1R, Eumelanin and Pheomelanin: Their role in determining the susceptibility to skin cancer. **2015**, *91*, 188-200.
37. Wei, C.-Y.; Zhu, M.-X.; Lu, N.-H.; Peng, R.; Yang, X.; Zhang, P.-F.; Wang, L.; Gu, J.-Y.J.O. Bioinformatics-based analysis reveals elevated MFSD12 as a key promoter of cell proliferation and a potential therapeutic target in melanoma. **2019**, *38*, 1876-1891.
38. Del Bino, S.; Duval, C.; Bernerd, F.J.I.j.o.m.s. Clinical and biological characterization of skin pigmentation diversity and its consequences on UV impact. **2018**, *19*, 2668.
39. Kawakami, A.; Fisher, D.E.J.L.i. The master role of microphthalmia-associated transcription factor in melanocyte and melanoma biology. **2017**, *97*, 649-656.
40. Wang, C.; Kocher, T.D.; Wu, J.; Li, P.; Liang, G.; Lu, B.; Xu, J.; Chen, X.; Wang, D.J.A. Knockout of microphthalmia-associated transcription factor (mitf) confers a red and yellow tilapia with few pigmented melanophores. **2023**, *565*, 739151.
41. Kim, D.-H.; Lee, J.; Ko, J.-K.; Lee, K.J.C.B. Melanophilin regulates dendritogenesis in melanocytes for feather pigmentation. **2024**, *7*, 592.
42. Yuan, Z.; Zhang, X.; Pang, Y.; Qi, Y.; Wang, Q.; Hu, Y.; Zhao, Y.; Ren, S.; Huo, L.J.A.A.B. Association analysis of melanophilin (MLPH) gene expression and polymorphism with plumage color in quail. **2023**, *66*, 131-139.
43. Zhang, H.; Wu, Z.; Yang, L.; Zhang, Z.; Chen, H.; Ren, J.J.T.F.J. Novel mutations in the Myo5a gene cause a dilute coat color phenotype in mice. **2021**, *35*, e21261.
44. O'Sullivan, T.N.; Wu, X.S.; Rachel, R.A.; Huang, J.-D.; Swing, D.A.; Matesic, L.E.; Hammer III, J.A.; Copeland, N.G.; Jenkins, N.A.J.P.o.t.N.A.o.S. dsu functions in a MYO5A-independent pathway to suppress the coat color of dilute mice. **2004**, *101*, 16831-16836.
45. Kidd, K.K.; Pakstis, A.J.; Donnelly, M.P.; Bulbul, O.; Cherni, L.; Gurkan, C.; Kang, L.; Li, H.; Yun, L.; Paschou, P.J.S.r. The distinctive geographic patterns of common pigmentation variants at the OCA2 gene. **2020**, *10*, 15433.
46. Vacher, J.; Bruccoleri, M.; Pata, M.J.I.J.o.M.S. Ostm1 from mouse to human: Insights into osteoclast maturation. **2020**, *21*, 5600.
47. Pandruvada, S.N.; Beauregard, J.; Benjannet, S.; Pata, M.; Lazure, C.; Seidah, N.G.; Vacher, J.J.M.; biology, c. Role of Ostm1 cytosolic complex with kinesin 5B in intracellular dispersion and trafficking. **2016**.
48. Watt, B.; van Niel, G.; Raposo, G.; Marks, M.S.J.P.c.; research, m. PMEL: a pigment cell-specific model for functional amyloid formation. **2013**, *26*, 300-315.
49. Hee, J.S.; Mitchell, S.M.; Liu, X.; Leonhardt, R.M.J.S.r. Melanosomal formation of PMEL core amyloid is driven by aromatic residues. **2017**, *7*, 44064.
50. Yoshida-Amano, Y.; Hachiya, A.; Ohuchi, A.; Kobinger, G.P.; Kitahara, T.; Takema, Y.; Fukuda, M.J.P.O. Essential role of RAB27A in determining constitutive human skin color. **2012**, *7*, e41160.
51. Jacobs, L.C.; Hamer, M.A.; Gunn, D.A.; Deelen, J.; Lall, J.S.; Van Heemst, D.; Uh,

H.-W.; Hofman, A.; Uitterlinden, A.G.; Griffiths, C.E.J.J.o.I.D. A genome-wide association study identifies the skin color genes IRF4, MC1R, ASIP, and BNC2 influencing facial pigmented spots. **2015**, *135*, 1735-1742.
52. Liu, F.; Visser, M.; Duffy, D.L.; Hysi, P.G.; Jacobs, L.C.; Lao, O.; Zhong, K.; Walsh, S.; Chaitanya, L.; Wollstein, A.J.H.g. Genetics of skin color variation in Europeans: genome-wide association studies with functional follow-up. **2015**, *134*, 823-835.
53. Twumasi, G.; Wang, H.; Xi, Y.; Qi, J.; Li, L.; Bai, L.; Liu, H.J.A. Genome-Wide Association Studies Reveal Candidate Genes Associated with Pigmentation Patterns of Single Feathers of Tianfu Nonghua Ducks. **2023**, *14*, 85.
54. Al Mahi, A.; Ablain, J.J.D.M.; Mechanisms. RAS pathway regulation in melanoma. **2022**, *15*, dmm049229.
55. Costin, G.-E.; Hearing, V.J.J.T.F.j. Human skin pigmentation: melanocytes modulate skin color in response to stress. **2007**, *21*, 976-994.
56. Naik, P.P.; Farrukh, S.N.J.S.p.; physiology. Influence of ethnicities and skin color variations in different populations: a review. **2022**, *35*, 65-76.
57. Batai, K.; Cui, Z.; Arora, A.; Shah-Williams, E.; Hernandez, W.; Ruden, M.; Hollowell, C.M.; Hooker, S.E.; Bathina, M.; Murphy, A.B.J.P.g. Genetic loci associated with skin pigmentation in African Americans and their effects on vitamin D deficiency. **2021**, *17*, e1009319.
58. Branicki, W.; Brudnik, U.; Draus-Barini, J.; Kupiec, T.; Wojas-Pelc, A.J.J.o.h.g. Association of the SLC45A2 gene with physiological human hair colour variation. **2008**, *53*, 966-971.
59. Huo, L.; Zhang, X.; Pang, Y.; Qi, Y.; Ren, S.; Wu, F.; Shang, Y.; Xi, J.J.T.J.o.P.S. Expression and Mutation of SLC45A2 Affects Iris Color in Quail. **2024**, *61*, 2024015.
60. Wang, L.-m.; Bu, H.-y.; Song, F.-b.; Zhu, W.-b.; Fu, J.-j.; Dong, Z.-j.J.C.B.; Molecular, P.P.A.; Physiology, I. Characterization and functional analysis of slc7a11 gene, involved in skin color differentiation in the red tilapia. **2019**, *236*, 110529.
61. Chen, Y.; Hu, S.; Mu, L.; Zhao, B.; Wang, M.; Yang, N.; Bao, G.; Zhu, C.; Wu, X.J.I.j.o.m.s. Slc7a11 modulated by POU2F1 is involved in pigmentation in rabbit. **2019**, *20*, 2493.
62. Arnoldi, A.; Tonelli, A.; Crippa, F.; Villani, G.; Pacelli, C.; Sironi, M.; Pozzoli, U.; D'Angelo, M.G.; Meola, G.; Martinuzzi, A.J.H.m. A clinical, genetic, and biochemical characterization of SPG7 mutations in a large cohort of patients with hereditary spastic paraplegia. **2008**, *29*, 522-531.
63. Yee, N.S.; Kazi, A.A.; Yee, R.K.J.C. Cellular and developmental biology of TRPM7 channel-kinase: implicated roles in cancer. **2014**, *3*, 751-777.
64. Shchagina, O.; Stepanova, A.; Mishakova, P.; Kadyshev, V.; Demina, N.; Bessonova, L.; Ionova, S.; Guseva, D.; Marakhonov, A.; Zinchenko, R.J.B. Common Variants in the TYR Gene with Unclear Pathogenicity as the Cause of Oculocutaneous Albinism in a Cohort of Russian Patients. **2024**, *12*, 2234.
65. Lai, X.; Wichers, H.J.; Soler-Lopez, M.; Dijkstra, B.W.J.C.A.E.J. Structure and

function of human tyrosinase and tyrosinase-related proteins. **2018**, *24*, 47-55.
66. Wagatsuma, T.; Suzuki, E.; Shiotsu, M.; Sogo, A.; Nishito, Y.; Ando, H.; Hashimoto, H.; Petris, M.J.; Kinoshita, M.; Kambe, T.J.C.b. Pigmentation and TYRP1 expression are mediated by zinc through the early secretory pathway-resident ZNT proteins. **2023**, *6*, 403.
67. Gissen, P.; Johnson, C.A.; Gentle, D.; Hurst, L.D.; Doherty, A.J.; O'Kane, C.J.; Kelly, D.A.; Maher, E.R.J.H.m.g. Comparative evolutionary analysis of VPS33 homologues: genetic and functional insights. **2005**, *14*, 1261-1270.
68. Graham, S.C.; Wartosch, L.; Gray, S.R.; Scourfield, E.J.; Deane, J.E.; Luzio, J.P.; Owen, D.J.J.P.o.t.N.A.o.S. Structural basis of Vps33A recruitment to the human HOPS complex by Vps16. **2013**, *110*, 13345-13350.
